# Encephalopathy: Cause, Pathogenesis, and Treatment

**DOI:** 10.1002/mco2.70802

**Published:** 2026-06-08

**Authors:** Shimeng Lv, Xia Zhong, Ruirui Shang, Linghui Kong, Yufei Huang, Yuexiang Ma, Jing Teng, Sheng Wei

**Affiliations:** ^1^ First Clinical Medical College Shandong University of Traditional Chinese Medicine Jinan China; ^2^ Institute of Child and Adolescent Health School of Public Health Peking University Beijing China; ^3^ College of Rehabilitation Medicine Shandong University of Traditional Chinese Medicine Jinan China; ^4^ Ruijin Hospital Affiliated to Shanghai Jiaotong University School of Medicine Shanghai China; ^5^ College of Traditional Chinese Medicine Shandong University of Traditional Chinese Medicine Jinan China; ^6^ Institute for Chinese Medicine and Brain Science/Key Laboratory of Traditional Chinese Medicine Classical Theory, Ministry of Education/Shandong Key Laboratory of Innovation and Application Research in Basic Theory of Traditional Chinese Medicine/Shandong Provincial Engineering Research Center for the Prevention and Treatment of Major Brain Diseases with Traditional Chinese Medicine Shandong University of Traditional Chinese Medicine Jinan China

**Keywords:** cause, clinical translation, encephalopathy, pathological mechanism, therapeutic potential

## Abstract

Encephalopathy refers to diffuse brain dysfunction caused by various systemic pathological processes such as systemic infections, metabolic disorders, and organ failure. This condition poses a formidable challenge in neurocritical care, with major subtypes encompassing sepsis‐associated encephalopathy, hepatic encephalopathy, hypoxic–ischemic encephalopathy, diabetic encephalopathy, uremic encephalopathy, and toxic encephalopathy. The current therapeutic landscape reveals a critical deficiency in effective neuroprotective interventions, highlighting an urgent need for novel treatment strategies. Small‐molecule compounds, particularly those derived from natural products, offer a promising therapeutic paradigm due to their multitarget capabilities and potential for network‐level modulation of pathogenic processes. However, advancement in this field remains constrained by several fundamental limitations: fragmented mechanistic insights, ill‐defined target networks, and insufficient clinical translation. To address these challenges, this review systematically synthesizes contemporary evidence to delineate the etiopathogenesis of these encephalopathies, with particular emphasis on the molecular mechanisms and cellular targets of small molecule drugs (especially natural products). Through a critical assessment of current research limitations, this review aims to establish a robust framework and provide forward‐looking perspectives to guide the development of targeted neuroprotective strategies and their clinical translation.

## Graphical abstract

Various encephalopathies (sepsis‐associated, hepatic, hypoxic–ischemic, diabetic, uremic, toxic) have incompletely elucidated pathogenesis, which severely restricts targeted therapy development. Small molecule drugs show unique multitarget potential but face toxicity, poor blood–brain barrier penetration and unclear specificity. Clarifying these mechanisms and targets holds great scientific value for drug development and clinical translation.

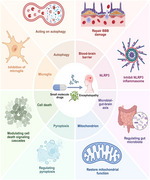

## 1 Introduction

Encephalopathy is a syndrome of diffuse brain dysfunction triggered by various systemic pathological factors (such as systemic infection, metabolic disturbances, or intoxication), posing a severe challenge in neurocritical care and multidisciplinary diagnosis and treatment. Although the initial causes of various encephalopathies such as sepsis‐associated encephalopathy (SAE), hepatic encephalopathy (HE), hypoxic–ischemic encephalopathy (HIE), diabetic encephalopathy (DE), uremic encephalopathy, and toxic encephalopathy differ, accumulating evidence suggests that they share common pathological pathways, ultimately resulting in neuronal injury or cognitive impairment. Although etiology‐specific management is the cornerstone of clinical practice for specific encephalopathies, targeted neuroprotective therapies that directly preserve neurons and improve the cerebral microenvironment remain a notable weakness.

Faced with these treatment challenges, small‐molecule compounds, notably natural products, are positioned as a highly promising therapeutic strategy against the complex pathological network of encephalopathies, given their diverse biological activities and strong drug development potential [1]. They possess the capability to simultaneously target multiple pathological processes, holding promise for achieving multidimensional modulation of neuroinflammation and other pathways. However, the development of this field still faces significant bottlenecks. For instance, existing research remains fragmented, lacking systematic integration of the etiology, mechanisms, and therapeutic strategies across different encephalopathies. Additionally, the target networks of small‐molecule drugs are still poorly defined. Finally, there is insufficient translation from basic research to clinical application. These collective challenges hinder the efficient development of targeted neuroprotective drugs.

To address these challenges, this review provides a comprehensive summary and analysis of six major encephalopathies: SAE, HE, HIE, DE, uremic encephalopathy, and toxic encephalopathy. It systematically discusses their causes and pathological mechanisms, with a particular emphasis on elucidating the molecular mechanisms and targets of small‐molecule drugs (especially natural products) in treatment strategies. By critically evaluating the limitations of existing research, this review also aims to establish a solid theoretical foundation and offer forward‐looking insights for clarifying complex neural interaction networks, identifying key therapeutic targets, and promoting subsequent basic research and clinical translation.

## 2 Sepsis‐Associated Encephalopathy

SAE is a brain dysfunction caused by diffuse sepsis, in which patients exhibit varying degrees of cognitive impairment, memory loss, and behavioral abnormalities [2]. The exact pathophysiology of SAE remains unclear, posing significant challenges for the development of targeted therapeutic strategies. Current clinical interventions for severe sepsis‐related events, such as the use of antibiotics, may inadvertently lead to secondary complications like epilepsy, cognitive changes, and the development of antibiotic resistance [3]. A deeper understanding of the pathogenesis of SAE is therefore critical for developing effective and safe treatments. Recent research has revealed that SAE involves a multifactorial pathogenesis, primarily characterized by blood–brain barrier (BBB) disruption, neuroinflammation, mitochondrial dysfunction, and glial cell activation. Elucidating these interconnected pathological processes not only offers a rationale for the clinical manifestations of SAE but may also uncover novel therapeutic targets.

### 2.1 Causes and Pathological Mechanisms

#### 2.1.1 BBB Injury

The exchange of chemicals between the cerebrospinal fluid and plasma is selectively regulated by the BBB, a dynamic interface. Tight connections between capillary endothelial cells, glial cells, the basement membrane, and pericytes compose it. By regulating the admission and departure of biological materials required for neurological and metabolic processes, the BBB plays a crucial part in preserving brain homeostasis. Disruption of the BBB allows neurotoxic blood‐derived debris, cells, and microbial pathogens to enter the brain, triggering inflammation, immune responses, and a variety of neurodegenerative pathways [4, 5].In sepsis, BBB damage is commonly observed. For example, in a lipopolysaccharide (LPS)‐induced SAE model, increased BBB permeability was found in the cerebral cortex, olfactory cortex, hippocampus, and thalamus of rats, leading to impaired vascular function and barrier integrity [6]. Postmortem brain tissue from sepsis patients also showed abnormal expression of junction proteins, indicating BBB damage [7]. When the BBB is compromised, the central nervous system (CNS) becomes more vulnerable to neurotoxic substances like free radicals, inflammatory mediators, and circulating white blood cells, which contribute to the progression of sepsis‐related brain injury [8, 9].

Mechanistic studies have identified key proteins involved in BBB disruption. For example, Annexin A1 (ANXA1) levels were reduced in septic mice, and its deficiency exacerbated cognitive impairment and decreased survival rates. ANXA1 deficiency also upregulated proinflammatory cytokines and adhesion molecules, worsened BBB damage, and altered tight junction protein expression and vascular endothelial growth factor (VEGF)‐A/VEGF‐R2 signaling [10]. Additionally, polymerase δ‐interacting protein 2 (Poldip2), a multifunctional protein involved in mitochondrial dynamics and DNA repair, mediates LPS‐induced BBB disruption through activation of nuclear factor kappa B (NF‐κB) and induction of cyclooxygenase‐2 (Cox‐2) and prostaglandin E2 [11]. Endotoxemia‐induced systemic inflammation also impairs endothelial Wnt/β‐catenin signaling by activating NF‐κB, leading to acute BBB disruption and neuroinflammation [12]. Caspase‐11 and gasdermin D (GSDMD) have also been implicated in BBB disruption and neuroinflammation, contributing to synaptic damage in the hippocampus [13, 14].

Therefore, the disruption of the BBB is a key pathological step in the onset and progression of SAE. Sepsis can compromise the structural and functional integrity of the BBB, increasing its permeability, which in turn facilitates the translocation of neurotoxic substances into the brain and triggers brain injury. However, current explanations for the mechanisms underlying BBB disruption remain fragmented. Most studies focus on individual molecules or pathways, lacking a systematic understanding of how these factors form a dynamic regulatory network. Additionally, the majority of research treats the BBB as a homogeneous entity, overlooking the structural and functional heterogeneity across different brain regions and vascular segments, as well as the specific responses of this heterogeneity in SAE. Finally, although some therapeutic strategies have shown efficacy in animal models, their translation to clinical practice faces significant challenges, such as how to achieve efficient and targeted drug delivery to the affected sites.

#### 2.1.2 Neuroinflammation

The inflammatory reaction that occurs inside the CNS as a result of several clinical insults such infection, trauma, ischemia, and toxins is referred to as neuroinflammation. The brain's innate immune cells are activated during this process, releasing chemokines and proinflammatory cytokines. For instance, when the BBB sustains biochemical or mechanical damage, endothelial cells become activated, express adhesion molecules, and release proinflammatory cytokines, thereby promoting the infiltration of peripheral immune cells such as monocytes and lymphocytes. These infiltrating blood cells further release inflammatory mediators, exacerbating the neuroinflammatory response. They act in synergy with microglia and astrocytes, leading to neuronal damage and synaptic dysfunction [15]. Activated microglia, key components of the brain's innate immune system, release inflammatory cytokines that cause neurotoxicity to nearby neurons, contributing to the development of various neurological disorders [16]. A critical player in the brain's immune response is NOD‐like receptor protein 3 (NLRP3), which detects exogenous pathogens and endogenous cell damage. Upon activation, NLRP3 forms inflammasomes, complexes that activate caspase‐1 [17], leading to the hydrolysis of proinflammatory cytokines like interleukin‐1β (IL‐1β) and inducing inflammatory cell death [18]. In an SAE‐related in vitro study, LPS‐induced activation of reactive oxygen species (ROS) and NLRP3 was observed [19]. Additionally, activation of NLRP3 in microglia under LPS stimulation leads to the production of microvesicles containing IL‐1β, contributing to synaptic defects [20]. The purinergic ligand‐gated ion channel 7 (P2×7) receptor, a purinergic ligand‐gated ion channel, also mediates NLRP3 inflammasome activation in microglia. In SAE models, LPS‐induced activation of this receptor exacerbates neuroinflammation, and targeting P2×7 can reduce neuroinflammation and prevent cognitive impairment [21, 22]. Furthermore, extracellular vesicles (EVs) derived from plasma of septic mice promote proinflammatory responses in microglia both in vitro and in vivo, with mechanisms partially mediated by Toll‐like receptor 7 (TLR7) in vitro and myeloid differentiation primary response 88 signaling in vivo [23]. Further research has identified additional molecules involved in neuroinflammation. Growth differentiation factor 15 (GDF15), a member of the transforming growth factor beta superfamily, is elevated in SAE mice and has been shown to alleviate cognitive and memory impairments targeted with antibodies. GDF15 reduces microglial inflammation and phagocytosis, thereby improving sepsis‐induced brain dysfunction [24, 25].

Neuroinflammation is a central pathological process in SAE, involving complex interactions among microglia, the NLRP3 inflammasome, the P2×7 receptor, and multiple signaling pathways. Existing research has not only preliminarily elucidated the initiation and amplification mechanisms of neuroinflammation but has also identified potential therapeutic targets for SAE. However, current understanding is largely derived from LPS‐induced acute inflammation models, which differs from human SAE that is primarily triggered by bacterial infections in clinical settings and frequently involves chronic and complex pathological processes. This discrepancy substantially limits the clinical translational value of relevant research findings. Furthermore, the current comprehension of neuroinflammatory mechanisms often remains confined to isolated, linear pathway descriptions. A systematic understanding of how key signaling nodes such as NLRP3 and P2×7 form interactive networks, engage in crosstalk, and dynamically regulate the inflammatory cascade in an integrated manner remains inadequate. Translating the numerous molecular targets identified in basic research into clinical interventions capable of efficiently crossing the BBB with high specificity remains a critical challenge for future research.

#### 2.1.3 Neuroplasticity

Neuroplasticity is a fundamental concept in neuroscience, describing the brain's ability to adapt and evolve by forming new neural connections. This adaptability is essential for cognitive functions such as memory, learning, and brain recovery following injury [26, 27, 28]. Neuroplasticity can be categorized into two types: structural and functional plasticity [29, 30]. Structural plasticity involves changes in the physical structure of the brain, such as axonal growth, dendritic spine formation, and the promotion of neurogenesis [31]. Functional plasticity, in contrast, refers to the brain's ability to alter synaptic strength, neurotransmitter function, and receptor activity without changing its physical structure [32].

Clinical research has shown that sepsis, especially when accompanied by neuroinflammation, can lead to decreased expression of synaptic plasticity‐related proteins in the cerebrospinal fluid of newborns [33]. Similar findings have been observed in preclinical studies. For instance, hippocampal transcriptomics and metabolomics analyses revealed abnormal synaptic plasticity in SAE [34], and LPS‐induced changes in synaptic plasticity‐related gene (PRG) (ARC and EGR1) expression were found in septic mice [35]. Subsequent studies suggested that high mobility group box 1 protein (HMGB1) plays a pivotal role in these changes by inducing microglial activation, abnormal synaptic pruning, and neuronal dysfunction, ultimately leading to cognitive impairment in SAE animal models [36].

The neurotransmitter serotonin (5‐hydroxytryptamine) has been implicated in the cognitive impairment associated with SAE [37]. Additionally, gamma‐aminobutyric acid (GABA), a key nonprotein amino acid involved in neuronal growth, synaptic communication, and mood regulation, plays an essential role in the brain's response to SAE [38]. In SAE, the N‐acetyltransferase 10 enzyme in hippocampal dentate gyrus neurons promotes GABAB receptor expression through mRNA acetylation, contributing to cognitive impairment [39]. Further studies have shown that active matrix metalloproteinases‐9 mediates remodeling of the perineural net, leading to reduced inhibitory and excitatory inputs to parvalbumin interneurons. This results in abnormal γ oscillations in the hippocampal CA1 region, driving the pathological progression of LPS‐induced SAE [40]. Brain‐derived neurotrophic factor (BDNF) plays a crucial role in neuroplasticity and is involved in several brain disorders. BDNF's involvement in SAE has drawn significant attention due to its impact on cognitive function [41]. Studies have shown that the hippocampus–prefrontal cortex (HPC–PFC) pathway is essential for improving cognitive function in septic mice [42]. Additionally, the downstream BDNF signaling pathway mediated by glutamate receptors is critical in connecting the HPC–PFC pathway with SAE [2].

Therefore, impaired neuroplasticity represents a potential core mechanism underlying SAE‐related cognitive dysfunction. Sepsis can induce multifaceted impairments in neuroplasticity, spanning molecular to neural circuit levels. However, current research in this field remains largely descriptive, with limited understanding of the causal and temporal relationships among these abnormalities, as well as their upstream regulatory mechanisms. For instance, how neuroinflammation, neurotransmitter imbalances, and altered neuroplasticity interact to form a vicious cycle remains an area requiring further investigation.

#### 2.1.4 Microbial–Gut–Brain Axis

The human gastrointestinal system hosts a diverse microbial population that plays a significant role in a wide range of biological processes, including development, aging, health maintenance, and disease. The gut microbiota's vast genetic and metabolic capacity is involved in regulating many physiological functions, and it has a profound impact on the CNS via the microbiota–gut–brain (MGB) axis [43]. This axis represents the bidirectional communication between gut microorganisms and the CNS. In the context of SAE, neuroinflammation and BBB disruption are key pathogenic mechanisms, and the gut plays a central role in both the onset and progression of sepsis and MODS. Dysbiosis, or an imbalance in the gut microbiota, may contribute to the development of SAE through the MGB axis. Sepsis‐induced gut dysfunction can exacerbate systemic inflammation, which, in turn, promotes distal organ damage via this axis [44].

Sepsis‐induced damage to the intestinal environment often leads to an overgrowth of harmful bacteria, triggering intestinal inflammation and disrupting mucosal immunity. For example, the polysaccharide components of Gram‐negative bacterial cell walls, particularly LPS, are recognized by TLR4, LPS‐binding proteins, and myeloid differentiation factor 2 on intestinal cells. This recognition activates downstream signaling pathways involving TIR domain‐containing adaptor proteins or the TRIF (Toll/IL‐1 receptor) pathway, which in turn activate NF‐κB and interferon regulatory factor 3, stimulating intestinal immune cells to release proinflammatory cytokines [45, 46]. LPS also causes the breakdown of epithelial cell junctions, leading to increased intestinal permeability, exacerbating systemic inflammation and promoting organ failure in sepsis [47, 48, 49, 50, 51].

The MGB axis not only involves microbial signals but also microbial metabolites, which can influence the CNS. Indole‐3‐propionic acid (IPA), a major tryptophan derivative produced by gut microbiota, has been shown to alleviate anxiety and spatial memory impairments in SAE models. IPA also prevents microglia from activating the NLRP3 inflammasome, a key player in neuroinflammation [52, 53]. Another important metabolite is 3‐indoleacetic acid, produced by gut bacteria, which protects against SAE by activating the aryl hydrocarbon receptor in microglia, thereby reducing neural and cognitive deficits [54]. Short‐chain fatty acids (SCFAs), including butyrate, propionate, and acetate, are produced by gut bacteria through fermentation of dietary fibers. SCFAs play an essential role in regulating the epithelial barrier, mucosal immunity, and systemic immune responses through mechanisms involving G protein‐coupled receptors and histone deacetylase inhibition [55]. However, in SAE, the levels of acetate and propionate are significantly reduced, accompanied by gut microbiota dysbiosis, particularly a decrease in bacteria producing SCFAs [56]. Disruption of gut microbiota and SCFA metabolism contributes to the pathological development of SAE, and supplementation with SCFAs has been shown to have neuroprotective effects by maintaining the integrity of the BBB [57]. Emerging evidence also suggests a potential link between nonhepatic hyperammonemia and SAE through the microbiota‐gut‐brain axis. Zhao et al. reported that sepsis‐induced dysbiosis leads to an increase in urease‐producing bacteria, which boosts ammonia synthesis and alters amino acid metabolism. This dysregulation results in elevated levels of aquaporins‐4 (AQP4) in astrocytes, facilitating the entry of ammonia into the brain. The accumulation of ammonia in the brain contributes to neuronal injury and compromised structural integrity of neurological fibers, a hallmark of SAE [58].

#### 2.1.5 Glial Cells

Glial cells are essential components of the CNS in all living organisms. As the complexity of the brain increases, glial cells have evolved, showing substantial growth in both number and function [59]. In the mammalian CNS, glial cells, including astrocytes, microglia, oligodendrocytes, and oligodendrocyte precursor cells, account for approximately half of all nerve cells, primarily supporting and nourishing neurons [60]. These cells play pivotal roles in brain function and pathology, including in the development of SAE.

Astrocytes, the most abundant and widespread glial cells in the brain, play a crucial role in maintaining brain homeostasis. They interact extensively with neurons and other glial cells, contributing to various brain functions such as nutrient support, ion balance, and neurotransmitter regulation [61]. In SAE, astrocytes are involved in the disease's pathological development. Specifically, IL‐11 derived from astrocytes has been shown to regulate astrocyte–microglial crosstalk in SAE through the NF‐κB signaling pathway [62]. Astrocytes also express a membrane‐bound water channel called AQP4, which is critical for water transport. In SAE, AQP4 exacerbates cognitive impairment by preventing Na_v_1.6‐mediated autophagy in astrocytes, worsening the overall neuronal dysfunction in SAE [63]. In addition, activation of hippocampal α2A adrenergic receptors has shown potential in protecting against SAE. The mechanism involves inhibiting the reactivity of astrocytes and reducing glutamate neurotoxicity, which ultimately helps prevent synaptic damage [64].

Microglia, the resident macrophages of the brain, are critical in regulating brain growth, maintaining neuronal networks, and mediating repair processes following injury. These cells are involved in both normal brain function and the pathogenesis of various neuroinflammatory diseases. In SAE, microglial activation is a significant factor in cognitive impairment. Microglia are responsible for clearing harmful agents such as microorganisms, protein aggregates, and other antigens that could damage the CNS [65]. Microglial polarization, a process where microglia take on different functional states depending on the environment, plays a crucial role in the pathophysiology of SAE [66]. Studies have shown that septicemia induces overexpression of the stimulator of interaction genes in microglia and macrophages, leading to synaptic loss, abnormal theta oscillations, and damage to long‐term potentiation. This process contributes to cognitive impairment through microglial activation and complement C1q regulation [67]. In addition, research by Chung et al. found that microglia contribute to neurocognitive impairment in SAE by phagocytosing C1q‐labeled synapses [68].

In summary, glial cells, particularly astrocytes and microglia, are central to the pathogenic progression of SAE. Their involvement in neuroinflammation, synaptic dysfunction, and BBB integrity highlights the critical role they play in the disease. Furthermore, targeting glial cells and their associated molecular pathways could provide novel therapeutic avenues for treating SAE and similar neuroinflammatory conditions.

#### 2.1.6 Mitochondrion

Mitochondria are central to many cellular processes, including energy production through oxidative phosphorylation. These organelles play crucial roles in cellular homeostasis, and disruptions in their function can have widespread impacts on systemic metabolism, aging, and overall health [69]. The complexity of mitochondrial function is essential in disease development and progression. Dysfunction in mitochondria is implicated in several common diseases, such as cancer, metabolic syndrome, neurodegenerative disorders, and cardiovascular diseases. Understanding how mitochondrial dysfunction contributes to illness is challenging due to its complex nature and the lack of clear phenotypic thresholds [70].

In sepsis, mitochondrial damage occurs through various mechanisms, and impaired mitochondria initiate multiple signaling pathways that exacerbate disease, especially within the CNS [71]. In the case of SAE, mitochondrial function in the hippocampus is severely impaired, leading to an increase in ROS production, which contributes to neuronal apoptosis, inflammation, and worsened cognitive deficits [72]. Key signaling pathways like the hypoxia‐inducible factor‐1α (HIF‐1α)/BCL2/adenovirus E1B interacting protein 3‐like pathways, which regulate processes such as cell survival, apoptosis, and autophagy, also contribute to mitochondrial damage and the progression of SAE [73]. Additionally, overexpression of heat shock proteins, specifically HSP8, can help alleviate cognitive decline in SAE by regulating mitochondrial biogenesis, homeostasis, and neuroinflammation [74]. Another study found that DCA could protect mitochondria and provide neuroprotection in SAE by inhibiting dynamin‐related protein 1 (DRP1) [75]. Fgr, a tyrosine kinase, has been implicated in mitochondrial dysfunction, and its inhibition in SAE models improved hippocampal mitochondrial function [76]. Furthermore, the redox‐related transcription factor nuclear factor‐E2‐related factor 2 (Nrf2) [77] may reduce sepsis‐induced brain damage by controlling mitochondrial homeostasis [78].

In summary, mitochondrial dysfunction represents a significant pathological mechanism in SAE, capable of triggering neuronal apoptosis and neuroinflammation, which ultimately contribute to cognitive impairment. Although existing studies have preliminarily established the central role of mitochondria in SAE and identified several potential interventional targets, the understanding of the dynamic changes in the mitochondrial quality control system during SAE and its interactions with other organelles remains to be further elucidated.

#### 2.1.7 Programmed Cell Death

Programmed cell death (PCD) is an essential biological process in health and disease. It is regulated by molecular mechanisms such as autophagy, pyroptosis, and ferroptosis. These mechanisms play pivotal roles in the pathological progression of SAE, and modulating PCD pathways can alleviate its severity [79, 80, 81, 82]

Pyroptosis is a form of inflammatory PCD, often initiated by inflammatory substances and executed by GSDMD. The primary features of pyroptosis include cell swelling, membrane rupture, and the release of cellular contents, which can lead to widespread inflammation and tissue damage [83]. In sepsis, pyroptosis plays a critical role in the immune response, being activated early in the infection to inhibit pathogen replication and facilitate pathogen removal. However, when the infection is not adequately controlled, pathogens can evade immune detection, leading to excessive pyroptosis, which amplifies the systemic inflammatory response and may result in organ failure and septic shock [84]. Pyroptosis also mediates the pathological processes associated with sepsis‐induced SAE. Both typical and atypical pyroptotic pathways contribute to cell damage induced by LPS [85]. The activation of GSDMD and the subsequent inflammatory cascade are crucial defense mechanisms against pathogens, as cell death initiated by pyroptosis can help clear intracellular infections [86]. However, in animal models of SAE, such as the cecal ligation puncture (CLP) model, GSDMD activation, along with upregulation of DRP1, mediates mitochondrial dysfunction, neuroinflammation, and neuronal and synaptic damage [87]. Furthermore, neuronal nuclear autophagy is activated in SAE, and inhibition of GSDMD mitigates this autophagic process, resulting in improved behavioral outcomes [88]. Further mechanistic investigations have shown that programmed death ligand 1 plays an important role in maintaining the transcriptional activity of p‐Y705–signal transducer and activator of transcription 3 (STAT3), which promotes GSDMD‐dependent net release in septic neutrophils, contributing to the development of SAE [89]. Additionally, in LPS‐induced SAE, the tripartite motif‐containing protein 45 exacerbates neuroinflammatory responses by facilitating microglial apoptosis through the Atg5/NLRP3 signaling axis [90].

A key therapeutic strategy for SAE is preventing excessive pyroptosis. Erbin, an interacting protein of ErbB2, inhibits the activity of the IRE1/XBP1s pathway in SAE, thereby reducing the influx of calcium into the cytoplasm and decreasing astrocyte pyroptosis [91]. Adipose‐derived stem cells (ADSCs) have garnered attention in regenerative medicine due to their pluripotency, ease of accessibility, and active paracrine activity [92]. In SAE, ADSCs play a significant therapeutic role, with ADSC‐derived exosomes providing neuroprotection by modulating the GSDMD signaling pathway, thereby improving cognitive impairment [93]. Additionally, JQ1, a tool compound used to study the role of bromodomain and extraterminal proteins, helps slow neuroinflammation in SAE. It protects the hippocampus, BBB, and neurons from damage by acting on the inflammasome‐dependent pyroptosis pathway [94].

Autophagy is a vital cellular process that helps maintain homeostasis by degrading and recycling damaged proteins and organelles. While autophagy generally serves as a protective mechanism, excessive autophagic flux or disruption of autophagic pathways can lead to cell death and contribute to disease progression [95]. In SAE, abnormal autophagy has been observed, with pathological manifestations [96], such as altered expression of microtubule‐associated protein light chain 3 (LC3)‐I and LC3‐II, which are key markers of autophagic activity [97]. When enhancing autophagic activity, transferrin receptor 1 (TFR1) can be degraded to inhibit ferroptosis, thereby improving cognitive function in SAE models [98]. Additionally, chaperone‐mediated autophagy (CMA), a selective form of autophagy, plays an important role in cellular homeostasis. In CLP mice, the expression of CMA‐related markers, such as lysosome‐associated membrane protein 2A and heat shock cognate 71 kDa protein, is significantly reduced, indicating dysregulated CMA activity during SAE [99]. Mitophagy, the selective degradation of damaged mitochondria, is another critical autophagic process that ensures mitochondrial and cellular homeostasis [100]. In SAE, promoting mitophagy can be a beneficial therapeutic approach. High concentrations of H_2_, referred to as HCH, have been shown to enhance mitophagy activity, which in turn helps reduce neuroinflammation and protect neuronal function in SAE [101].

Ferroptosis is a distinct form of iron‐dependent, nonapoptotic cell death, triggered by the peroxidation of phospholipids. It is regulated by various cellular metabolic processes, including redox homeostasis, iron metabolism, mitochondrial activity, and the metabolism of sugars, lipids, and amino acids, alongside multiple disease‐associated signaling pathways. Ferroptosis is implicated in several organ damages and degenerative diseases [102, 103, 104]. It is closely linked to sepsis, a condition that affects multiple organs and systems by disrupting the body's response to infection. Severe stress during sepsis can induce disturbances in ion, lipid, and energy metabolism. One hallmark of ferroptosis is the disruption of iron metabolism and the accumulation of iron‐dependent lipid peroxides, which contributes to cellular damage. This nonapoptotic form of cell death [105] is also involved in sepsis‐induced brain damage. In the CLP model of sepsis, ferroptosis is associated with a reduction in glutathione peroxidase 4 (GPX4) levels, increased transferrin and malondialdehyde levels, and mitochondrial atrophy [106]. Furthermore, sepsis induces high expression of nuclear enriched transcript 1 derived from serum exosomes, which may exacerbate ferroptosis by regulating the miR‐9‐5p/TFR and glutamic oxaloacetic transaminase 1 axis, thereby worsening SAE [107].

**FIGURE 1:**
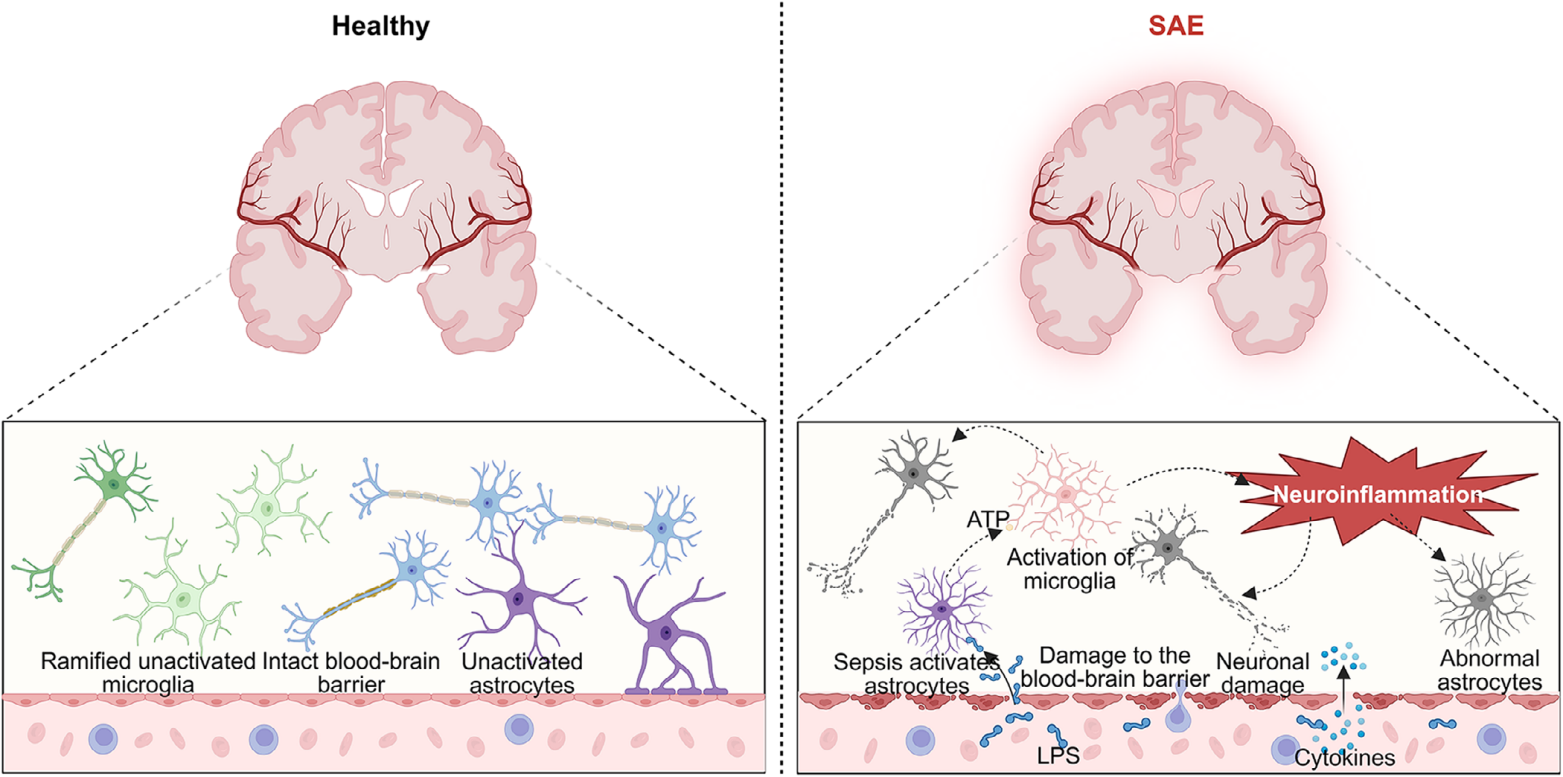
The difference between a healthy brain and a septic brain. After the BBB is damaged in sepsis, LPS, and other cytokines enter the brain, overactivating glial cells and causing damage to neurons. *Abbreviations*: BBB, blood–brain barrier; LPS, lipopolysaccharide.

**FIGURE 2:**
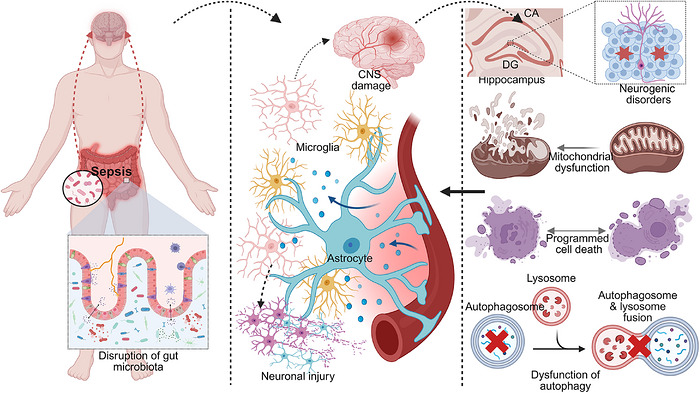
Pathological mechanism of SAE. In addition to intestinal barrier degradation, sufferers of sepsis experience intestinal microbiota disorders that worsen their systemic response. Certain cytokines reach the CNS, where they cause a number of severe damage to neurons, along with cell death and mitochondrial malfunction. *Abbreviations*: CNS, central nervous system; SAE, sepsis‐related encephalopathy.

Several therapeutic strategies targeting ferroptosis have shown potential in ameliorating SAE. For example, Maresin1 has demonstrated therapeutic effects by reducing hippocampal neuroinflammation through the activation of the solution carrier family 7 member 11 (SLC7A11)/GPX4 ferroptosis signaling pathway, leading to improvements in cognitive impairment associated with SAE [108]. Acetaminophen, a commonly used analgesic and antipyretic drug [109], has been shown to inhibit cerebral ferroptosis in septic mice by regulating GPX4 and ferroptosis suppressor protein 1 [110]. In addition, propofol counteracts ferroptosis by activating the Nrf2/heme oxygenase‐1 (HO‐1) axis, thereby preventing brain damage caused by sepsis [111]. Irisin also inhibits hippocampal ferroptosis through the Nrf2/GPX4 signaling pathway, improving the inflammatory microenvironment in SAE [112] (Figures 1 and 2).

PCD, particularly pyroptosis, autophagy, and ferroptosis, plays a critical role in mediating brain injury during sepsis and contributes significantly to the pathophysiology of SAE. While these forms of PCD represent promising therapeutic targets, their precise molecular mechanisms and distinct roles in SAE pathogenesis remain to be fully elucidated. Moreover, the interplay between different PCD pathways and their regulatory impact on SAE progression in a coordinated manner continues to be an active focus of investigation.

### 2.2 Treatment

#### 2.2.1 Flavonoids Natural Products

Plants contain secondary metabolites called flavonoids, which are natural molecules with various pharmacological properties, including anti‐inflammatory, anticancer, antioxidant, and vascular protective effects. These compounds are found in many plant types, including fruits and vegetables [113]. One such flavonoid is quercetin, which occurs naturally in foods like apples, almonds, and tomatoes. Quercetin has demonstrated excellent safety when used to treat respiratory and cardiovascular disorders due to its anti‐inflammatory and antioxidant properties [114]. Recent studies have shown that quercetin can improve symptoms caused by CLP, such as changes in body temperature, neurological severity scores, and learning and memory impairments in mice. It also inhibits the C–X–C motif chemokine ligand 2 (CXCL2)/C–X–C motif chemokine receptor 2 (CXCR2) pathway, blocking interactions between microglia and neurons and providing neuroprotective effects against SAE [115]. Genistein, a major isoflavone found in soy products [116], improves cognitive function in septic rats by reducing inflammation and oxidative stress, while also activating the Nrf2 pathway [117]. Morin, another flavonoid found in fruits such as mulberries [118], also offers therapeutic effects in SAE by exerting anti‐inflammatory and antioxidant effects, improving survival rates, and enhancing the behavioral function of sepsis animal models [119].

Puerarin is a natural isoflavone extracted from the root of *Pueraria montana* (Lour.) Merr., a plant known for its cardiovascular protective properties in traditional Chinese medicine [120]. In SAE, puerarin inhibits the classic pyroptosis pathway mediated by NLRP3/caspase‐1/GSDMD, alleviates BBB damage [121], and modulates the protein kinase B 1 (AKT1) pathway in microglia, exerting anti‐inflammatory effects in SAE [122]. However puerarin may have embryotoxic effects. A study by Chen et al. found that puerarin (at concentrations of 2.5, 5, and 10 mol/L)‐induced apoptosis in mouse embryonic vesicle ICM cells, reducing embryonic development and survival [123]. It also causes abnormal apoptosis in early embryos through a caspase‐dependent mechanism, leading to cell loss and hindering postimplantation development [124]. Therefore, although puerarin demonstrates significant therapeutic potential by targeting multiple pathological pathways in SAE, its reported embryotoxicity poses a potential obstacle to clinical translation. Consequently, a comprehensive toxicological assessment is essential before considering puerarin for therapeutic applications. For instance, systematic in vivo toxicological studies are still required to determine the no‐observed‐adverse‐effect level and establish a safe therapeutic window in relevant SAE animal models.

#### 2.2.2 Alkaloids Natural Products

Alkaloids are a class of naturally occurring nitrogen‐containing organic compounds that are widely distributed in plants, animals, and microorganisms. They often have complex molecular structures, are typically alkaline, and exhibit a variety of biological activities. These characteristics make alkaloids an important source of compounds for drug development. Gelsevirine, an alkaloid isolated from *Gelsemium elegans* Benth, a traditional Chinese herbal medicine, possesses multiple pharmacological properties [125]. Gelsevirine has been shown to increase the survival rate of mice with SAE, improve cognitive function, inhibit glial cell activation, and alleviate inflammation. Its mechanism may involve the inhibition of microglial pyroptosis [126]. Palmatine, a nonartificial quinoline alkaloid isolated from various botanical families, is a classic anti‐inflammatory compound in the Chinese Pharmacopoeia [127]. In SAE mice, palmatine improves brain function, restores intestinal barriers, reduces neuroinflammation, and shows positive regulatory effects on brain diseases, fecal metabolism, and gut microbiota [128]. Berberine, the primary bioactive component of *Coptis chinensis* Franch, has various beneficial pharmacological effects [129]. In SAE, berberine shows promise by targeting the HMGB1/RAGE signaling pathway to prevent the decline of A1 astrocytes and newborn neurons under stress in microglia [130]. Additionally, it suppresses the NF‐κB signaling pathway in CLP models, reducing inflammation and neuronal cell death [131].

#### 2.2.3 Polyphenol Natural Products

Polyphenols are a widespread class of compounds found in nature, known for their biocompatibility, biological adhesion, antioxidant, and antibacterial properties [132]. Natural polyphenols play a crucial role in the treatment of sepsis [133, 134] and also contribute to the management of SAE. Resveratrol, first isolated from the roots of *Veratrum album* L, is a natural plant toxin produced by plants to protect them from environmental stress and pathogen invasion. It exhibits multiple pharmacological activities [135]. Resveratrol alleviates cognitive impairment in SAE by preventing ER stress and preserving ER function in microglia [136]. Additionally, it inhibits the NLRP3/IL‐1β signaling pathway in microglia, improving spatial memory in SAE mice [137]. Catechin hydrate, a polyphenolic compound primarily found in plants, alleviates multiorgan damage caused by sepsis, including brain injury. It achieves this by inhibiting RasGRP1, a novel activator of macrophage proinflammatory responses [138]. Polydatin, the primary bioactive component isolated from the roots of *Reynoutria japonica* Houtt, alleviates SAE by upregulating anti‐inflammatory and mitochondrial protective mechanisms mediated by sirtuin 1 (SIRT1) [139]. Americanin B (AMEB), isolated from the seed coat of *Vernicia fordii* (Hemsl.) Airy Shaw, has garnered attention for its anti‐inflammatory and antioxidant properties. In SAE, AMEB targets the NLRP3 protein to prevent LPS‐induced pyroptosis in mice with septic encephalopathy [140]. Although natural polyphenols have shown potential in alleviating SAE, there are still significant limitations in current research. Most of the evidence is based on preclinical animal models, which have pathological and physiological differences from human SAEs, and their effectiveness and safety still need to be clinically validated. Second, the pharmacokinetic properties of polyphenols, such as bioavailability, BBB permeability, and in vivo metabolic processes, have not been fully studied. In addition, how to translate basic research results into clinical strategies (including timing of administration, dosage form selection, and individualized treatment plans) remains a key challenge for the future.

#### 2.2.4 Terpenoids Natural Products

Terpenoids are the most diverse class of natural products, with various biological activities, diverse structures, and complex functions. These compounds have gained widespread attention due to their broad range of effects [141]. Patchouli, also known as *Pogostemon cablin* (Blanco) Benth, a member of the Orchidaceae family, has been used in traditional Chinese medicine since the Eastern Han Dynasty [142]. β‐patchoulene (β‐PAE), one of the active natural tricyclic sesquiterpenes in essential oil isolated from *Pogostemon cablin* (Blanco) Benth, has anti‐inflammatory, antioxidant, and hepatoprotective effects [143]. In SAE, β‐PAE significantly reduces sepsis‐induced neuroinflammation and microglial activation, reversing cognitive decline and improving peripheral immune function. This neuroprotective effect may be mediated by the activation of the Sirt1/Nrf2/HO‐1 pathway [144]. β‐Elemene (β‐ELE), derived from *Curcuma aromatica* Salisb [145], is known for its antitumor and anti‐inflammatory biological activity [146]. In both in vivo and in vitro SAE models, β‐ELE can effectively alleviate the pathological symptoms in SAE mice. It inhibits the Ras‐related C3 botulinum toxin substrate 1 (RAC1)/mixed‐lineage kinase 3 (MLK3)/p38 signaling pathway in microglia and shows promise as a potential candidate for treating SAE [147].

#### 2.2.5 Terpenoids Natural Products

Saponins are a type of natural product with diverse structures and functions. Characterized by their unique triterpenoid/steroid skeleton and glycoside modification, saponins are multitarget compounds with anti‐inflammatory, antitumor, and immune‐regulatory properties. They are widely used in various applications due to these beneficial effects. *Panax ginseng* C.A. Mey has been used as an excellent medicinal herb in China for thousands of years. It contains ginsenoside Rg1, an active ingredient with anti‐inflammatory, antioxidant, and neuroprotective properties [148]. Ginsenoside Rg1 can inhibit the activation of NF‐κB and mitogen‐activated protein kinases (MAPK) signaling pathways [149] and also regulates the noncanonical Beclin 1‐independent autophagy pathway, playing a neuroprotective role [150]. Gypenosides have neuroprotective and other effects [151]. In the CLP‐induced mouse SAE model, gypenoside XLIX can improve the survival rates, slow down BBB damage, and alleviate oxidative stress, cell apoptosis, and neuroinflammation. Its mechanism of action involves the targeted regulation of peroxisome proliferator‐activated receptor (PPAR)‐α [152].

#### 2.2.6 Dexmedetomidine

Unlike the complex composition of natural products, dexmedetomidine, a highly selective α2‐adrenergic receptor agonist, serves as a unique and potent pharmacological tool for precise investigation of SAE treatment mechanisms due to its sedative, analgesic, and potential neuroprotective properties [153]. Studies have reported that dexmedetomidine treats SAE by suppressing neuroinflammation via α2A‐adrenergic receptors in astrocytes, while also improving neuroinflammation‐induced deficits in hippocampal neurogenesis through α2A‐adrenergic receptors in neural stem cells [154]. Moreover, dexmedetomidine improves sleep quality and reduces sleep fragmentation in SAE mice through its sedative effect, thereby promoting the clearance of abnormally deposited α‐synuclein (α‐Syn) in the brain. This process ultimately alleviates anxiety and depression‐like psychiatric symptoms and improves SAE prognosis [155].

#### 2.2.7 Other Types of Compounds

**FIGURE 3:**
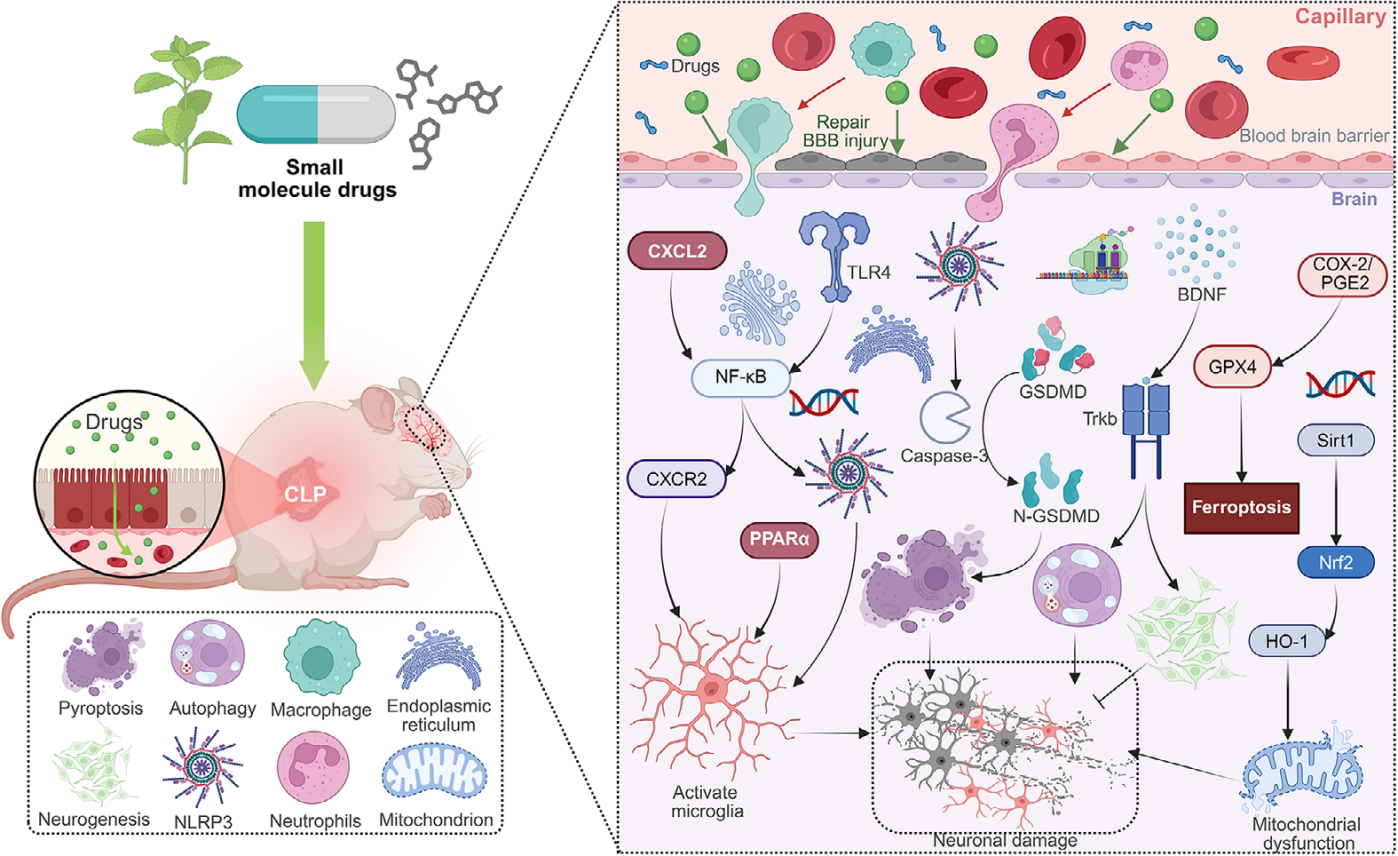
The molecular mechanism of small molecule drugs in SAE. *Abbreviations*: CLP, cecal ligation and puncture; CXCL2, C–X–C motif chemokine ligand 2; NF‐κB, nuclear factor‐kappa B; CXCR2, C–X–C motif chemokine receptor 2; PPAR, peroxisome proliferator‐activated receptor; TLR4, Toll‐like receptor 4; BDNF, brain‐derived neurotrophic factor; GSDMD, gasdermin D; Cox‐2, cyclooxygenase‐2; GPX4, glutathione peroxidase 4; HO‐1, heme oxygenase‐1; Nrf2, nuclear factor erythroid 2‐related factor 2; Sirt1, sirtuin 1; NLRP3, NOD‐like receptor protein 3.

Compounds of other categories have also been investigated for SAE treatment. Propofol has been reported to exert therapeutic effects through modulation of the Nrf2/HO‐1 signaling pathway [111]. Metformin ameliorates sepsis‐associated neurological damage by regulating the gut microbiota and metabolites in septic rats [156]. Magnesium sulfate administration is correlated with improved survival in SAE, which may be attributed to its multitarget regulation of inflammatory responses and immune modulation [157]. In summary, beyond natural products, dexmedetomidine and other synthetic compounds also exhibit therapeutic potential against SAE through various mechanisms. However, most of these findings are derived from preclinical studies, and their efficacy and safety in humans remain highly uncertain. Furthermore, substantial species differences exist between animal models and human SAE patients in terms of etiological complexity, intensity of pathophysiological responses, as well as structural and functional aspects of the CNS (Figure 3 and Table 1).

**TABLE 1:** Information on natural products and compounds in SAE.

Natural product/compound	CAS No.	Molecular formula	Modeling method	Dose	Molecular mechanism	References
Quercetin	117‐39‐5	C_15_H_10_O_7_	In vivo: CLP In vitro: LPS	In vivo: 30, 50 mg/kg In vitro: 5 µM	Inhibition of CXCL2/CXCR2 pathway blocks the interaction between microglia and neurons	[115]
Genistein	446‐72‐0	C_15_H_10_O_5_	CLP	15 mg/kg	Reduce inflammatory response and oxidative stress, as well as activate the Nrf2 pathway	[117]
Morin	480‐16‐0	C_15_H_10_O_7_	LPS	50 mg/kg	Anti‐inflammatory and antioxidant effects	[119]
Puerarin	3681‐99‐0	C_21_H_20_O_9_	In vivo: CLP In vitro: LPS	In vivo: 150 mg/kg In vitro: 400 µM	Inhibit the classic pyroptosis pathway mediated by NLRP3/caspase‐1/GSDMD and alleviate BBB damage	[121]
			LPS	In vivo: 40, 80 mg/kg In vitro: 25, 50, 100, 200 µM	Regulating the AKT1 pathway in microglia to exert anti‐inflammatory effects against SAE	[122]
Gelsevirine	38990‐03‐3	C_21_H_24_N_2_O_3_	CLP	1, 5, 10 mg/kg	Inhibition of STING signaling pathway mediated pyroptosis in microglia	[126]
Palmatine	3486‐67‐7	C_21_H_22_NO_4_^+^	LPS	2.5 mg/kg	Regulating the microbiota–gut–brain axis	[128]
Berberine	2086‐83‐1	C_20_H_18_NO_4_^+^	In vivo: CLP In vitro: LPS	In vivo: 10 mg/kg In vitro: 5 µM	Regulating the HMGB1/RAGE signaling pathway	[130]
			CLP	In vivo: 50 mg/kg In vitro: 0–100 µM	Inhibition of hippocampal NF‐κB/LCN2 pathway reduces inflammation and neuronal cell death	[131]
Resveratrol	501‐36‐0	C_14_H_12_O_3_	LPS	250 mg/kg	Inhibiting ER stress and maintaining the homeostasis of ER function in microglia	[136]
			In vivo: CLP In vitro: LPS	In vivo: 10, 30 mg/kg In vitro: 15, 30 µM	Inhibition of NLRP3/IL‐1β signaling pathway in microglia	[137]
Catechin hydrate	88191‐48‐4	C_15_H_16_O_7_	In vivo: CLP In vitro: LPS	In vivo: 40, 80 mg/kg In vitro: 50 µg/mL	Inhibit RasGRP1	[138]
Polydatin	27208‐80‐6	C_20_H_22_O_8_	CLP	45 mg/kg	Upregulation of Sir1 mediated neuroinflammation inhibition and mitochondrial function protection	[139]
Americanin B	77053‐44‐2	C_27_H_24_O_9_	LPS	In vivo: 20 mg/kg In vitro: 10 µM	Preventing pyroptosis and brain damage by blocking the activation of NLRP3 inflammasome	[140]
β‐Patchoulene	−	−	CLP	0.2, 1 mg/kg	Activation of Sirt1/Nrf2/HO‐1 pathway mediates neuroprotective effects	[144]
β‐Elemene	−	−	In vivo: CLP In vitro: LPS	In vivo: 10, 20, 40 mg/kg In vitro: 1, 5, 25 µM	Inhibition of RAC1/MLK3/p38 signaling pathway activation and inflammatory response in hippocampus	[147]
Ginsenoside Rg1	22427‐39‐0	C_42_H_72_O_14_	LPS	In vivo: 200 mg/kg In vitro: 10, 30 µM	Activation of NF‐κB and MAPK signaling pathways	[149]
			CLP	40, 200 mg/kg	Regulating the noncanonical Beclin 1‐independent autophagy pathway	[150]
Gypenoside XLIX	94987‐08‐3	C_52_H_86_O_21_	In vivo: CLP In vitro: LPS	In vivo: 40 mg/kg In vitro: 10 µM	Targeted regulation of PPAR‐α	[152]
Dexmedetomidine	113775‐47‐6	C_13_H_16_N_2_	In vivo: CLP In vitro: LPS	In vivo: 20 µg/kg In vitro: 10 0.1, 1, 100 µM	Inhibiting neuroinflammation via the α2A‐adrenergic receptor on astrocytes	[154]
			LPS	50 µg/kg	Reduce α‐synuclein deposition	[155]
Propofol	2078‐54‐8	C_12_H_18_O	LPS	50, 75, 100 mg/kg	Activation of Nrf2/HO‐1 pathway inhibits ferroptosis	[111]
Metformin	657‐24‐9	C_4_H_11_N_5_	CLP	100 mg/kg	Regulating gut microbiota and metabolites	[156]

*Abbreviations*: BBB, blood–brain barrier; CLP, cecal ligation and puncture; CXCL2, C–X–C motif chemokine ligand 2; CXCR2, C–X–C motif chemokine receptor 2; GSDMD, gasdermin D; HMGB1, high mobility group box 1 protein; HO‐1, heme oxygenase‐1; IL‐1β, interleukin‐1β; LPS, lipopolysaccharide; MAPK, mitogen‐activated protein kinases; MLK3, Mixed‐lineage kinase 3; NF‐κB, nuclear factor kappa B; NLRP3, NOD‐like receptor protein 3; Nrf2, nuclear factor‐E2‐related factor 2; RAC1, Ras‐related C3 botulinum toxin substrate 1; SAE, sepsis‐associated encephalopathy; Sirt1, sirtuin 1.

## 3 Hepatic Encephalopathy

HE is broadly defined as brain dysfunction caused by hepatic insufficiency and/or portosystemic shunting, manifesting as a spectrum of neurological or psychiatric abnormalities ranging from subclinical alterations to coma. While hyperammonemia represents its core pathological mechanism, the specific molecular pathways involve multifaceted interactions that have not yet been fully elucidated. This incomplete understanding contributes to a scarcity of effective treatment strategies. Current interventions remain substantially limited in terms of timeliness, efficacy, and prevention of irreversible neurological damage, underscoring the urgent need for novel therapeutic approaches [158].

### 3.1 Causes and Pathological Mechanisms

#### 3.1.1 Ammonia Poisoning

While the intestines are the primary site of ammonia production from protein digestion, amino acid deamination, and bacterial urease activity, a healthy liver with an intact urea cycle regulates systemic concentrations, thus keeping blood ammonia levels within normal limits. Impaired liver detoxification and portosystemic shunting prevent the effective clearance of intestinally produced ammonia, leading to hyperammonemia. As a result, this elevated blood ammonia directly or indirectly causes neurological injury, constituting a key driving force in HE pathogenesis. For instance, upon entering cells via rapid diffusion, ammonia binds with intracellular protons to form ammonium, leading to transient intracellular alkalinization. As ammonium is transported across membranes, the intracellular pH gradually normalizes. The kinetics of this pH recovery are influenced by multiple factors, including the rate and concentration of ammonia entry along with the expression and function of potassium channels and ammonia transporters. Additionally, dysfunction of connexin hemichannels is considered to disrupt metabolic communication between astrocytes and neurons, thereby contributing to cerebral dysfunction [159, 160].

Recurrent episodes of ammonia‐induced HE can also lead to neuronal cell loss. Excessive ammonia affects neuroglial cells, particularly astrocytes, resulting in morphological changes and functional abnormalities. It inhibits glutaminase, which is an enzyme mainly present in neurons, leading to reduced replenishment of the synaptic glutamate pool and impaired neurotransmission. In animal models of HE with chronic hyperammonemia, mitochondrial dysfunction, impaired respiratory chain enzyme activity, and mitochondrial swelling have been observed in multiple cell types, including astrocytes. These mitochondrial damages, combined with the high energy demand of ammonia detoxification (converted to glutamine) and enhanced Na^+^/K^+^‐ATPase activity, collectively cause ATP depletion, increased ROS, and impaired cerebral energy metabolism, which in turn lead to severe neuronal injury [160, 161, 162, 163, 164, 165, 166, 167, 168, 169, 170].

The neurotoxicity associated with chronic hyperammonemia originates from dysregulation of the N‐methyl‐d‐Aspartic acid (NMDA) receptor, which in turn induces neuronal damage by disrupting the nitric oxide–cyclic guanosine monophosphate pathway and triggering a cascade of events including bioenergetic crisis (ATP depletion and mitochondrial dysfunction) and oxidative stress [171, 172, 173].Furthermore, studies have revealed that hyperammonemia directly disrupts both GABAergic and glutamatergic systems, triggering brain region‐specific excitatory‐inhibitory imbalance, impairing neuron–glia interactions, and compromising BBB function [171, 174, 175, 176, 177, 178]. In the context of HE‐related motor dysfunction, hyperammonemia has been found to induce a transient increase in circulating IL‐17. This cytokine activates IL‐17 receptors in the cerebellum, leading to oxidative stress and BBB disruption. The resulting increase in barrier permeability facilitates further entry of IL‐17 into cerebellar tissue, which subsequently activates microglia and ultimately impairs motor coordination by potentiating GABAergic neurotransmission in the cerebellum [179].

These multifaceted mechanisms collectively constitute the core pathological pathway of hyperammonemia‐induced brain injury. However, current research remains predominantly focused on the preclinical level, failing to adequately reflect the complex clinical scenario involving dynamic fluctuations in ammonia levels and their synergistic effects with other hepatotoxins. Moreover, there is still insufficient understanding of the upstream events and key molecular switches triggered during the early phase of ammonia entry into cells that initiate signaling cascades.

#### 3.1.2 Neuroinflammation

Neuroinflammation is not only involved in the pathogenesis and progression of SAE but also exerts a significant impact on HE. For instance, neuroinflammatory factors (IL‐1β) impair synaptic plasticity by altering the membrane expression of α‐amino‐3‐hydroxy‐5‐methyl‐4‐isoxazolepropionic acid receptors and NMDA glutamate receptors, thereby leading to learning and memory impairments. Additionally, neuroinflammation can cause astrocyte dysfunction, which makes the GABA transporter 3 (GAT‐3) operate in reverse. This reverses GAT‐3 function to increase extracellular GABA levels and enhance inhibitory neurotransmission, ultimately triggering dysfunctions in motor coordination and learning [180]. In addition, systemic inflammation exacerbates the occurrence and progression of HE by promoting the release of proinflammatory cytokines (IL‐1β and IL‐6). These cytokines can not only directly induce astrocytic edema but also activate microglia, disrupt the BBB, and promote neuroinflammatory responses, thereby enhancing the neurotoxicity of ammonia and exacerbating the neuropsychiatric symptoms of HE. Particularly in cases of liver cirrhosis complicated with chronic infection, persistent inflammatory stimulation further drives the progression of HE severity and depressive symptoms [181]. The elevation and activation of IL‐17 receptor levels in microglia of rats with hyperammonemia contribute to the enhancement of cerebellar neuroinflammation [182]. Moreover, EVs in rats with hyperammonemia can cause hippocampal neuroinflammation (activation of NF‐κB and IL‐1 β production), impairing cognitive function in normal rats [183]. In summary, inflammation, particularly neuroinflammation, contributes to the pathogenesis of HE through multiple pathways, most notably by amplifying ammonia‐induced neurotoxicity. Therefore, interventions targeting neuroinflammation represent a crucial therapeutic strategy for HE.

#### 3.1.3 Manganese

Manganese is one of the most abundant transition metals in the earth's crust and an essential trace element for the human body. The liver is one of the primary organs for manganese metabolism and excretion, with manganese excreted via bile. When manganese intake is excessive or liver function is impaired, manganese accumulates in the liver, leading to toxicity [184]. In the context of HE, studies have shown that manganese deposition contributes to the development of HE [185]. In mechanistic studies, it has been found that manganese toxicity can potentially interfere with the communication between astrocytes and neurons at multiple levels, particularly by disrupting the glutamine–glutamate cycle, which in turn impairs neuronal metabolism and neurotransmission function [186]. In rat astrocyte models and animal models, manganese has been shown to downregulate the expression of glutamate–aspartate transporter and glutamate transporter 1, thereby inducing behavioral and motor function abnormalities [187]. Additionally, within astrocytes, manganese toxicity primarily induces oxidative and nitrosative stress, alters mitochondrial membrane potential, impairs mitochondrial function, and leads to the occurrence of astrocyte swelling, inflammation, and cerebral edema [188, 189].

#### 3.1.4 Other Types

Bile acids are products of cholesterol metabolism, synthesized in the liver via the activity of cytochrome P450 in hepatocytes. Levels of bile acids increase in the blood of patients with end‐stage liver disease due to the disruption of the enterohepatic circulation. Bile acids have been detected in the brain tissue of HE rats induced by bile duct ligation; this accumulation of bile acids further induces neuroinflammation. For example, it contributes to the progression of neurological dysfunction in HE by mediating the expression of Takeda G protein‐coupled receptor 5 and regulating neuroinflammation [190, 191, 192]. Chronic liver injury initially leads to elevated systemic levels of 17β‐estradiol (E2) and chenodeoxycholic acid. These substances subsequently activate their respective receptors, thereby downregulating the expression of key transport proteins—organic cation transporter 1, OCT2, and organic cation/carnitine transporter 2—at the BBB. The impaired function of these transporters reduces cerebral uptake of carnitine and acetylcarnitine, resulting in their decreased levels in the brain. Consequently, mitochondrial function in the brain is compromised, leading to disrupted energy metabolism and ultimately contributing to the pathogenesis of HE [193]. In the study of biomarkers for HE, it was found that glutamine and albumin in cerebrospinal fluid have the potential to serve as key biomarkers for evaluating the severity of neurological damage in patients with acute liver failure [194].

To conclude, the pathogenesis of HE is a complex process involving the synergistic effects of multiple factors, grounded in liver failure and portal‐systemic shunting. Among these, ammonia toxicity constitutes the core link: gut‐derived ammonia cannot be effectively cleared by the liver, leading to hyperammonemia that directly or indirectly impairs the nervous system. Neuroinflammation acts as a key “accelerator”: systemic inflammation and neuroinflammation themselves not only can independently damage the CNS but also exert a significant synergistic effect with ammonia toxicity, amplifying its neurodamaging role. Furthermore, the abnormal deposition of manganese in the brain, the neurotoxicity of bile acids, and the consequent cerebral energy metabolism crisis collectively form a multifaceted pathogenic network of HE. Based on these well‐defined pathological links, current therapeutic practices and research frontiers should aim to break this pathogenic cycle, so as to improve patients' neurological function and enhance their quality of life.

### 3.2 Treatment

#### 3.2.1 Natural Products

Natural products exhibit broad prospects in the treatment of HE, with their mechanisms of action mainly centered on its core pathological links. Naringenin, a prominent natural flavonoid compound, is mainly found in tomatoes, citrus fruits, and other fruits. Its ameliorative effect on the progression of HE is attributed to its regulation of the Jun N‐terminal kinase (JNK)/Bax/caspase‐8 apoptotic pathway [195]. Among other flavonoids, hesperidin mediates its therapeutic effects against HE through regulation of the NLRP3/Sirt1/forkhead box O transcription factor (FOXO) signaling pathway [196]. Curcumin has been shown to reduce cerebral edema, intracranial pressure, blood ammonia levels, and BBB permeability following hepatic and extrahepatic injury [197]. Taxifolin exerts hepatoprotective and neuroprotective functions via its antioxidant and anti‐inflammatory activities, along with inhibition of excessive ammonia release, ultimately reversing neurobehavioral impairments [198]. The natural monoterpenoid menthol demonstrates significant protective effects on both the liver and brain; it suppresses the expression of inflammatory factors, reduces neuronal death in the hippocampal CA1 region and dentate gyrus, alleviates astrocytic swelling, and thereby improves learning and spatial memory in HE rats [199].

Berberine not only shows certain therapeutic potential in SAE but also has been reported to exert effects in HE. It alleviates the behavioral and molecular abnormalities of HE by inhibiting the NLRP3 inflammasome pathway [200]. Additionally, berberine improves spatial learning and memory abilities in cirrhotic rats by enhancing BBB function, alleviating oxidative stress, and reducing neuroinflammation. It may thus serve as a potential therapeutic agent for improving cirrhotic cognitive impairment [201]. Although berberine has demonstrated neuroprotective effects in HE, there are reports of gastrointestinal discomfort, diarrhea, and abdominal pain in some diabetic patients treated with 500 mg of berberine three times daily for 13 weeks. However, these adverse reactions are transient and resolve after treatment [202]. Therefore, future research on berberine could explore novel drug delivery systems to improve its oral bioavailability and reduce gastrointestinal irritation. Second, dose optimization studies should be conducted to identify the minimum effective dose while ensuring efficacy, thereby lowering the incidence of adverse reactions.

#### 3.2.2 Compounds

Rifaximin, a nonabsorbable antibiotic, exhibits a favorable safety profile due to its low systemic absorption [203]. In minimal HE (MHE), rifaximin has been demonstrated to mitigate neuroinflammation in rats with mild liver injury by attenuating elevated C–C motif chemokine ligand 2 (CCL2) levels, monocyte infiltration, microglial activation, and tumor necrosis factor‐α (TNF‐α) elevation, while concurrently improving cognitive function [204]. Furthermore, the combination of rifaximin with lactulose modulates gut microbiota metabolism and preserves intestinal barrier integrity, thereby enhancing cognitive performance in MHE rats [205]. Additionally, dexmedetomidine effectively reversed sleep disturbances and neurobehavioral alterations in MHE rats while alleviating brain tissue damage. This neuroprotective mechanism may be partially attributed to the activation of α2‐adrenergic receptors, attenuation of neuroinflammatory responses, and inhibition of microglial activation along with the NLRP3/caspase‐1 signaling pathway [206]. Montelukast, one of the most widely used leukotriene modifiers, is a selective leukotriene receptor antagonist currently indicated for asthma prophylaxis and chronic treatment, relief of allergic rhinitis symptoms, and acute prevention of exercise‐induced bronchoconstriction [207]. It has demonstrated therapeutic potential in HE models by upregulating phosphatidylinositol 3‐kinase (PI3K)/Akt expression, thereby suppressing caspase‐3 activity as well as TNF‐α and NF‐κB activation [208].

Mirabegron is a selective β3‐adrenergic receptor agonist. In HE, it exerts significant hepatoprotective and neuroprotective effects on thioacetamide‐induced HE rat models by enhancing antioxidant defense, inhibiting pathological inflammatory and apoptotic pathways, and upregulating neuroprotective signaling pathways [209]. Some studies have explored the therapeutic effects of combining chemical compounds with natural products for HE, and found that compared with monotherapy, the combination of gallic acid and metformin can significantly ameliorate HE‐related complications. This combined regimen enhances their respective antioxidant, anti‐inflammatory, and antiapoptotic effects through a synergistic action [210]. However, when used in combination with natural products, close attention should be paid to the potential effects of drugs on body metabolism, and their pharmacokinetic interactions and safety profiles should be systematically evaluated. This is to develop rational medication strategies, thereby maximizing the synergistic therapeutic effects while minimizing the risk of adverse reactions (Table 2).

**TABLE 2:** Information on natural products and compounds in HE.

Natural product/compound	CAS No.	Molecular formula	Modeling method	Dose	Molecular mechanism	References
Naringenin	67604‐48‐2	C_15_H_12_O_5_	Thioacetamide	100 mg/kg	Regulating the apoptotic signaling pathway	[195]
Hesperidin	520‐26‐3	C_28_H_34_O_15_	Thioacetamide	100 and 200 mg/kg	Regulating the NLRP3/Sirt1/FOXO signaling pathway	[196]
Curcumin	458‐37‐7	C_21_H_20_O_6_	Acetaminophen	300 mg/kg	Reduce cerebral edema, intracranial pressure, blood ammonia, and BBB permeability following intrahepatic and extrahepatic injury	[197]
Taxifolin	24198‐97‐8	C_15_H_12_O_7_	Thioacetamide	50 and 100 mg/kg	Antioxidant and anti‐inflammatory effects, as well as inhibition of excessive release of ammonia	[198]
Menthol	1490‐04‐6	C_10_H_20_O	Thioacetamide	10 mg/kg	Inhibit oxidative stress, neuroinflammation, and neuronal death	[199]
Berberine	2086‐83‐1	C_20_H_18_NO_4_+	Thioacetamide	30 and 100 mg/kg	Inhibition of NLRP3 inflammasome pathway	[200]
				10, 30 and 60 mg/kg	Improve BBB function, oxidative stress, and neuroinflammatory response	[201]
Rifaximin	80621‐81‐4	C_43_H_51_N_3_O_11_	CCl_4_	20 mg/kg	Reduce neuroinflammation	[204]
Dexmedetomidine	113775‐47‐6	C_13_H_16_N_2_	Thioacetamide	20 and 40 µg/kg	Activate the α2‐adrenergic receptor, alleviate the neuroinflammatory response, and inhibit microglia as well as the NLRP3/caspase‐1 signaling pathway	[206]
Montelukast	158966‐92‐8	C_35_H_36_ClNO_3_S	Thioacetamide	5 and 10 mg/kg	Upregulation of PI3K/Akt expression, thereby inhibiting caspase‐3 expression and TNF‐α and NF‐κB activity	[208]
Mirabegron	223673‐61‐8	C_21_H_24_N_4_O_2_S	Thioacetamide	10 mg/kg	Upregulation of neuroprotective signaling pathways, protection of antioxidant defense and alleviation of neuroinflammation and apoptosis	[209]

*Abbreviations*: Akt, protein kinase B; BBB, blood–brain barrier; HE, hepatic encephalopathy; NF‐κB, nuclear factor kappa B; NLRP3, NOD‐like receptor protein 3; PI3K, phosphatidylinositol 3‐kinase; Sirt1, sirtuin 1; TNF‐α, tumor necrosis factor‐α.

## 4 Hypoxic–Ischemic Encephalopathy

HIE is a major cause of neurological dysfunction and long‐term poor prognosis in term neonates. Although therapeutic hypothermia has become one of the commonly used treatment approaches and has been proven to provide certain neuroprotective effects, its overall efficacy remains limited, and the improvement in infants’ prognosis has not met expectations. The global incidence of HIE shows significant geographical disparities: it is approximately one to eight cases per 1000 live births in developed countries, while in resource‐limited regions, this figure can be as high as 26 per 1000. Currently, the precise pathophysiological mechanisms of this disease have not yet been fully elucidated, which directly restricts the development of targeted therapeutic strategies. Therefore, exploring novel interventions that can exert a synergistic effect with therapeutic hypothermia has become a key research direction and urgent need for improving the clinical outcomes of HIE [211].

### 4.1 Causes and Pathological Mechanisms

#### 4.1.1 Neuroinflammation

Neuroinflammation serves a key function in the pathogenesis and progression of SAE, HE, and HIE. In HIE, monocytes promote the acute inflammatory response and may undergo long‐term transformation into pathological microglia following LPS/hypoxia–ischemia (HI)‐induced injury in neonates [212]. Additionally, in inflammation‐sensitized HIE, microglia exhibit a proinflammatory phenotype and promote the recruitment and activation of neutrophils to the brain by upregulating chemokines and adhesion molecules [213]. During the progression of HI‐induced white matter injury, dysmyelination, abnormal synaptic development, and behavioral alterations are observed in the periventricular white matter region. Meanwhile, the pathogenesis and progression of this injury are closely associated with NLRP3 inflammasome activation, microglial M1/M2 polarization, and inflammatory cytokine release [214]. Transient receptor potential vanilloid 1 (TRPV1) is a nonselective cation channel that is widely expressed in astrocytes and microglia within the CNS. By activating the Janus kinase 2 (JAK2)/STAT3 signaling pathway and the NLRP3 inflammasome, it promotes astrocyte activation and IL‐1β release, thereby exacerbating brain injury and neurobehavioral deficits induced by neonatal HI [215]. In HIE, the gut microbiota also has a potential association with neuroinflammation. When the neonatal gut is dominated by pathogenic bacteria such as *Escherichia coli*, it “preactivates” the systemic and cerebral immune systems. This leads to a more intense proinflammatory response in the brain (marked increases in TNF‐α and IL‐1β) during HI, and exacerbates the activation of immune cells such as microglia, thereby expanding the scope of cerebral edema and injury [216]. After HI injury, gut dysbiosis activates the LPS/TLR4 signaling pathway, inducing intestinal inflammation and dysfunction, exacerbating the systemic inflammatory response, and ultimately worsening synaptic and cognitive impairments in a rat model of Neonatal hypoxic–ischemic brain damage (HIBD) [217]. Targeting neuroinflammation holds potential for treating HIE; for instance, inhibiting TLR4 reduces oxidative stress‐induced injury, decreases the activation of ferroptosis, and alleviates neuroinflammation following HIBD [218]. PPAR‐γ exerts neuroprotective effects in hypoxic–ischemic white matter injury by regulating microglial polarization, inhibiting NF‐κB‐mediated neuroinflammation, and activating antioxidant pathways, thereby promoting myelin repair and neurological function recovery [219].

Taken together, neuroinflammation is the core pathological mechanism of HIE. Microglia polarize into a proinflammatory phenotype via signaling pathways such as NLRP3 inflammasome and JAK2/STAT3, and interact with the MGB axis—activated by gut dysbiosis through the LPS/TLR4 pathway. These interactions collectively exacerbate brain injury and neurological dysfunction. Targeting these neuroinflammatory pathways (e.g., TLR4, PPAR‐γ) shows potential therapeutic prospects. However, in HIE mechanism research, understanding of the dynamic process of microglial polarization and the specific mechanism of bidirectional communication in the MGB axis remains incomplete. Future studies need to further clarify the specific pathological role of neuroinflammation in HIE and explore the regulatory effects on key targets in neuroinflammation.

#### 4.1.2 Mitochondrion

Mitochondria are organelles enclosed by a double membrane, present in most eukaryotic cells, and serve as the structure responsible for energy production in cells. In HIE, mitochondria play a crucial role: for instance, HI directly induces mitochondrial energy metabolism failure, leading to ATP depletion. Meanwhile, it triggers the explosive production of large amounts of ROS and reactive nitrogen species within mitochondria, specifically peroxynitrite generated via reverse electron transport in Complex I and reaction with nitric oxide. These strong oxidants cause severe oxidative damage to mitochondrial proteins, lipids, and DNA, disrupt mitochondrial dynamics, and ultimately result in complete energy collapse, as well as trigger the program of cell apoptosis or necrosis [220]. MicroRNAs are short RNA molecules that play a central role in regulating gene expression, and they are involved in mediating processes related to mitochondria, neuroinflammation, and HIE. For example, in neonatal HI‐induced brain injury, miR‐210 acts as a key regulator of proinflammatory activation in microglia by reprogramming mitochondrial function [221]. A study found significant age‐dependent differences in the response of the neonatal rat brain to HI injury, particularly in terms of mitochondrial function. Compared with Day 3 postnatal rats (which simulate preterm infants), Day 11 postnatal rats (which simulate term infants) are more sensitive to injury, exhibiting severe mitochondrial dysfunction. This dysfunction is characterized by mitochondrial swelling, loss of membrane potential, decreased activity of key tricarboxylic acid cycle enzymes and respiratory chain complexes, as well as a significant reduction in oxygen consumption capacity [222]. Regulation of mitochondria also plays a role: as an illustration, studies have shown that mitochondrial‐targeted antioxidants exert neuroprotective effects in HIE by alleviating ROS bursts and stabilizing mitochondrial membrane potential [223]. Meanwhile, liposome‐delivered miR‐9‐5p alleviates HI‐induced brain injury by targeting zinc finger and BTB domain‐containing protein 20. This process inhibits the interaction between Kelch‐like ECH‐associated protein 1 (Keap1) and Nrf2, promotes Nrf2 nuclear translocation, enhances the antioxidant response, and improves mitochondrial function [224].

Additionally, Bcl‐2/adenovirus E1B 19 kDa interacting protein 3 (BNIP3)‐mediated mitophagy alleviates HIBD in neonatal rats. It achieves this by activating the P62/Keap1/Nrf2 pathway, maintaining iron and redox homeostasis, and inhibiting ferroptosis [225]. Mitochondrial dysfunction represents a central pathological mechanism in HIE. Interventions targeting mitochondrial function, including the use of mitochondria targeted antioxidants or modulation of pathways involving molecules such as BNIP3, have been proven to improve mitochondrial integrity, enhance antioxidant defenses, and demonstrate neuroprotective effects in animal models. Although research progress has been made, translating these mitochondrially targeted therapies from effective animal models to safe and efficient clinical treatments remains a critical challenge. Particularly, resolving issues related to BBB penetration and achieving cell specific targeting constitute the core problems demanding immediate solutions.

#### 4.1.3 Programmed Cell Death

PCD is not only a common pathway for neurological damage in SAE but also a key event in the pathological progression of HIE. Among PCD patterns, the sequential or synergistic activation of ferroptosis and pyroptosis forms the characteristic damage profile of this disease. In a study, by integrating bioinformatics analysis, machine learning, and experimental validation, researchers analyzed, identified, and confirmed that TNF, IL‐1β, and TLR2 are three core pyroptosis‐related hub genes in HIE [226]. Microglia‐mediated NLRP3/caspase‐1/GSDMD pyroptosis signaling pathway exerts a pivotal role in the pathological progression of neonatal HIE [227]. Additionally, the pyroptosis signaling pathway also mediates the process by which other factors induce or exacerbate HIE. Absent in melanoma 2 can promote neuronal pyroptosis via the caspase‐1/GSDMD pathway, thereby exacerbating the pathological damage of HIE [228]. Pyruvate kinase M2 is a key enzyme in glucose metabolism and is also closely associated with inflammatory responses; it can also promote hippocampal neuronal pyroptosis in HIBD through the NLRP3/caspase‐1/GSDMD pathway [229]. Meanwhile, targeted regulation of pyroptosis holds potential for HIE treatment: for instance, it has been reported that inhibiting cyclic GMP‐AMP synthase (cGAS) alleviates neonatal HIE by regulating microglial polarization and pyroptosis [230].

Ferroptosis has also been explored in the context of HIE. Asphyxia can affect iron metabolism in neonates with HIE, leading to a significant increase in nonprotein‐bound iron [231]. Neonatal HI induces decreased expression of GPX4 and glutathione in brain tissue, along with reduced antioxidant capacity and increased production of ROS. The acidic environment induced by HI can lead to the accumulation of free Fe^2^^+^ in brain tissue, which is consistent with the pathogenesis and progression of ferroptosis [232]. Furthermore, ferroptosis also mediates therapeutic responses in HIE: recent studies have shown that fibrinogen alpha chain‐derived peptide 12 inhibits ferroptosis and ameliorates HIBD by specifically targeting peroxiredoxin 1 [233]. Nuclear protein 1 (NUPR1) is a key regulator of ferroptosis; it counteracts ferroptosis in HIE, making it a potential therapeutic target for alleviating disease‐related neuronal damage [234].

PCD is a central mechanism in HIE‐induced neurological damage, where coordinated ferroptosis and pyroptosis create a unique pathological profile. Current limitations in understanding the interplay and shared regulation among PCD pathways hinder development of multitarget therapies. Future efforts should focus on deciphering core PCD cross‐talk, particularly between ferroptosis and pyroptosis, and advancing multitarget strategies toward clinical application.

### 4.2 Treatment

#### 4.2.1 Natural Products

Reports have demonstrated the therapeutic effects of flavonoid natural products in HIE. Echinatin, derived from *Glycyrrhiza inflata*, exerts its therapeutic effect on HIE by inhibiting the TLR4/NF‐κB pathway and alleviating neuroinflammation and cell apoptosis [235]. Another flavonoid natural product, myricetin, exerts a protective effect against HI‐induced injury by alleviating oxidative stress and cell apoptosis via the Nrf2 signaling pathway [236]. Carthamin yellow has been shown to alleviate brain injury in an experimental model of HIBD potentially by inhibiting the Nrf2/Keap1 pathway to reduce hippocampal neuronal ferroptosis [237]. Terpenoids also contribute to the treatment of HIE. Catalpol is an iridoid glycoside isolated from* Rehmannia glutinosa*, and it alleviates neuronal injury caused by HIBD by inhibiting ferroptosis‐related signaling pathways [238]. α‐Pinene is a natural monoterpenoid compound that exerts a protective effect against behavioral deficits induced by neonatal hypoxia by inhibiting neuroinflammation [239]. Perillyl alcohol, in turn, reduces neuronal injury in HIE by counteracting oxidative stress, alleviating inflammation, and inhibiting apoptosis via the Nrf2/Keap1 pathway [240].

Phenolic acid natural products are a class of important bioactive components, and chlorogenic acid—obtained from coffee—is one of them. Chlorogenic acid treatment reduces cerebral infarct volume in neonatal rats after HI, alleviates cerebral edema, improves tissue structure, and inhibits HI‐induced neuronal apoptosis by mitigating inflammation and oxidative stress [241].  Salvianolic acid C, a natural compound derived from *Salvia miltiorrhiza*, exhibits potential neuroprotective properties by reducing neuronal damage through suppression of oxidative stress and inflammatory responses [242]. Protocatechuic acid exerts a potent protective effect against ferroptosis‐mediated injury in HIE, and its neuroprotective mechanism is closely associated with regulating ferroptosis via the HIF‐1α/VEGFA signaling axis in cerebral microvascular endothelial cells [243]. Similarly, gallic acid treatment suppresses excessive ROS production and inflammatory cytokine release in microglia. Furthermore, it mitigates neuroinflammation and neuronal loss in HIBD rats, while also ameliorating motor and cognitive deficits [244]. Punicalagin is a polyphenolic compound obtained from *Punica granatum L*.; it significantly reduces cerebral infarction, neuroinflammation, and excessive autophagy induced by HIE, thereby exerting short‐term and long‐term neuroprotective effects. This mechanism is achieved by activating the AKT/FOXO4 pathway [245].

Natural products such as flavonoids, terpenoids, and phenolic acids demonstrate neuroprotective potential in HIE models through multitarget mechanisms including anti‐inflammatory, antioxidant, and antiferroptotic activities, primarily via modulating TLR4/NF‐κB signaling and ferroptosis. However, current research remains largely preclinical, lacking systematic assessment of molecular interactions, pharmacokinetics, and potential toxicity. Future studies should focus on elucidating synergistic mechanisms, optimizing bioavailability, and promoting translational development to advance these natural compounds as viable HIE interventions.

#### 4.2.2 Compounds

Beyond regulating circadian rhythms, melatonin also exhibits neuroprotective potential in HIE. Studies have demonstrated that melatonin maintains glymphatic system function by inhibiting the activity of cyclin‐dependent kinase 5 (CDK5) after HI and enhancing the interaction between AQP4 and α‐Syn [246]. Furthermore, melatonin‐induced activation of Notch homolog 1 (Notch1) signaling and rapid modulation of Sirt3 may contribute to neuronal survival during ischemic events [247]. At the mitochondrial level, melatonin targets the sodium‐calcium exchanger 1 via the PTEN‐induced putative kinase 1 (PINK1)/Parkin pathway to suppress excessive mitophagy [248]. Its neuroprotective effects also involve regulation of autophagy via the AMP‐activated protein kinase (AMPK)/mammalian target of rapamycin (mTOR) pathway [249]. Notably, combination therapy with melatonin and URB447 exhibits synergistic neuroprotection in HIE models, not only improving neurodevelopmental outcomes but also mitigating both gray and white matter injury [250].

Dexmedetomidine has been proven to exert neuroprotective effects in SAE. In HIE, its protective mechanism involves the synergistic action of multiple pathways. First, dexmedetomidine can induce the polarization of neurotoxic A1 astrocytes to neuroprotective A2 astrocytes, thereby activating the BDNF signaling pathway and promoting hippocampal neurogenesis. This ultimately reduces neuronal damage and cognitive impairment in neonates with HIBD [251]. Additionally, it can restore the survival and maturation capabilities of damaged oligodendrocyte progenitor cells and promote myelin regeneration [252]. Meanwhile, by upregulating miR‐148a‐3p to inhibit the STAT/Junonji domain‐containing protein 3 (JMJD3) signaling axis, Dexmedetomidine effectively reduces pyroptosis in hippocampal astrocytes, thus alleviating brain damage caused by cerebral hypoxia–ischemia in neonatal rats [253].

Atorvastatin exerts its neuroprotective effect by activating the BDNF pathway, thereby inhibiting neuronal apoptosis induced by HIBD and reducing brain injury in neonatal rats [254]. Furthermore, atorvastatin further demonstrates its therapeutic potential in HIE by promoting the proinflammatory‐to‐anti‐inflammatory phenotypic switch of microglia in neonatal rats via the Wnt/β‐catenin pathway [255]. Meanwhile, edaravone, a potent oxygen free radical scavenger, alleviates mast cell degranulation and neuroinflammation following HI injury through the ROS/stromal interaction molecule 1 (STIM1) pathway [256] (Table 3).

**TABLE 3:** Information on natural products and compounds in HIE.

Natural product/compound	CAS No.	Molecular formula	Modeling method	Dose	Molecular mechanism	References
Echinatin	34221‐41‐5	C_16_H_14_O_4_	In vivo: hypoxic–ischemic brain damage In vitro: oxygen‐glucose deprivation	In vivo: 1, 2.5, and 5 mg/kg In vitro: 2, 4, and 8 µM	Inhibition of TLR4/NF‐κB pathway	[235]
Myricetin	529‐44‐2	C_15_H_10_O_8_	In vivo: hypoxic–ischemic brain damage In vitro: CoCl_2_	In vivo: 25 mg/kg In vitro: 100 and 200 µM	Reduce oxidative stress and cell apoptosis through the Nrf2 signaling pathway	[236]
Carthamin yellow	1401‐20‐3	C_42_H_43_O_22_	Hypoxic–ischemic brain damage	40 mg/kg	Inhibition of Nrf2/Keap signaling pathway	[237]
Catalpol	2415‐24‐9	C_15_H_22_O_10_	In vivo: hypoxic–ischemic brain damage In vitro: oxygen‐glucose deprivation	In vivo: 2.5, 5, 10, and 20 mg/kg In vitro: 25–200 µM	Inhibiting signaling pathways related to ferroptosis	[238]
α‐pinene	2437‐95‐8	C_10_H_16_	Hypoxia induction	5 and 10 mg/kg	Inhibit inflammation	[239]
Perillyl alcohol	536‐59‐4	C_10_H_16_O	In vivo: Rice–Vannucci In vitro: glucose deprivation and hypoxia/reperfusion	In vivo: 25, 50 and 100 mg/kg In vitro: 50 µM	Regulating the Nrf2/Keap1 pathway to combat oxidative stress, alleviate inflammation, and inhibit apoptosis	[240]
Chlorogenic acid	1049703‐62‐9	C_16_H_18_O_9_	In vivo: Rice–Vannucci In vitro: oxygen‐glucose deprivation	In vivo: 150, 300, and 600 mg/kg In vitro: 100, 200, 300, and 400 µM	Activate Sirt1 to regulate Nrf2/NF‐κB signaling pathway	[241]
Salvianolic acid C	115841‐09‐3	C_26_H_20_O_10_	Hypoxic–ischemic brain damage	15 mg/kg	Inhibit oxidative stress and inflammation	[242]
Protocatechuic acid	99‐50‐3	C_7_H_6_O_4_	In vivo: Rice–Vannucci In vitro: oxygen–glucose deprivation/reoxygenation	In vivo: 50, 100, and 200 mg/kg In vitro: 9, 200, 27, and 81 µM	Regulation of HIF‐1α/VEGFA axis in brain microvascular endothelial cells on iron‐induced apoptosis	[243]
Gallic acid	149‐91‐7	C_7_H_6_O_5_	In vivo: hypoxic–ischemic brain damage In vitro: oxygen–glucose deprivation/reoxygenation	In vivo: 50 mg/kg In vitro: 0 and 40 µM	Reduce neuroinflammation and neuronal loss	[244]
Punicalagin	65995‐63‐3	C_48_H_28_O_30_	In vivo: Rice–Vannucci In vitro: glucose deprivation/reoxygenation	In vivo: 10 and 20 mg/kg In vitro: 10 and 20 µM	Regulating the AKT/FOXO4 pathway	[245]
Melatonin	8041‐44‐9	C_13_H_16_N_2_O_2_	Rice–Vannucci	5, 10, and 20 mg/kg	Inhibit the activity of CDK5 post‐HI to enhance the interaction between AQP4 and α‐Syn, thereby maintaining glymphatic function	[246]
			Cerebral hypoxia–ischemia	15 mg/kg	Melatonin induced Notch1 signaling pathway and regulation of Sirt3	[247]
			Hypoxic ischemic	10 mg/kg	Weakening the activation of PINK1/Parkin‐dependent mitochondrial autophagy pathway	[248]
			Hypoxia ischemia	25 mg/kg	Regulating the AMPK/mTOR pathway	[249]
Dexmedetomidine	113775‐47‐6	C_13_H_16_N_2_	Rice–Vannucci	25 µg/kg	Promoting hippocampal neurogenesis through BDNF/TrkB/CREB signaling pathway	[251]
			Rice–Vannucci	25 µg/kg	Promote the occurrence of oligodendrocytes and improve myelin formation	[252]
			In vivo: cerebral hypoxic ischemia In vitro: oxygen‐glucose deprivation	In vivo: 0.1 mg/kg In vitro: 1 µM	Upregulation of miR‐148 a‐3p inhibits apoptosis of hippocampal astrocytes, thereby suppressing the STAT/JMJD3 axis	[253]
Atorvastatin	110862‐48‐1	C_33_H_35_FN_2_O_5_	In vivo: hypoxic–ischemic brain damage In vitro: oxygen glucose deprivation	In vivo: 10 mg/kg In vitro: 1 µM	Activate the cAMP/PKA/p‐CREB/BDNF pathway	[254]
			In vivo: hypoxic–ischemic brain damage In vitro: oxygen glucose deprivation	In vivo: 10 mg/kg In vitro: 0.1, 1, and 10 µM	Promoting the proinflammatory/anti‐inflammatory phenotype transformation of neonatal rat microglia through the Wnt/β‐catenin pathway	[255]
Edaravone	89‐25‐8	C_10_H_10_N_2_O	In vivo: hypoxic–ischemic In vitro: oxygen glucose deprivation	In vivo: 3, 6, 9, and 18 mg/kg In vitro: 80 µM	Adjusting the ROS/STIM1 signaling pathway	[256]

*Abbreviations*: Akt, protein kinase B; AMPK, AMP‐activated protein kinase; AQP4, aquaporin 4; BDNF, brain‐derived neurotrophic factor; CDK5, cyclin‐dependent kinase 5; CREB, cAMP response element‐binding protein; FOXO4, forkhead box O4; HIE, hypoxic–ischemic encephalopathy; HIF‐1α, hypoxia‐inducible factor‐1α; mTOR, mammalian target of rapamycin; NF‐κB, nuclear factor kappa B; Nrf2, nuclear factor‐E2‐related factor 2; PINK1, PTEN‐induced putative kinase 1; ROS, reactive oxygen species; STIM1, stromal interaction molecule 1; TLR4, Toll‐like receptor 4; TrkB, tropomyosin receptor kinase B.

Although these studies have shed light on the potential mechanisms of action of various compounds in HIE, numerous unknowns remain. For instance, the crosstalk between different pathways and their specific regulation at different stages of development remain unclear; most evidence is derived from animal models, and their efficacy and safety in human neonates still need to be validated; the optimal timing of combination therapy and its impact on long‐term neurodevelopmental outcomes also await systematic evaluation. Future research should aim to deeply dissect the dynamic changes in molecular networks during the pathological process of HIE, facilitate multicenter clinical translational studies, and explore cell type‐specific therapeutic strategies, with the aim of providing more precise and effective neuroprotective therapies for neonates with HIE.

## 5 Diabetic Encephalopathy

DE is a severe complication induced by diabetes mellitus, belonging to the category of metabolic encephalopathies. Its core manifestation is cognitive impairment, with the most prominent feature being the decline in learning and memory abilities. With the global prevalence of diabetes mellitus, the incidence of DE has shown a concurrent upward trend. However, its exact pathogenesis remains under investigation to date, making it difficult to achieve fundamental breakthroughs in the development of targeted drugs [257].

### 5.1 Causes and Pathological Mechanisms

#### 5.1.1 Neuroinflammation

Neuroinflammation holds a critical position in the pathological progression of DE. Hyperglycemia can activate key inflammatory pathways such as NLRP3 to continuously activate microglia and astrocytes in the brain, promote the release of proinflammatory cytokines including IL‐6, TNF‐α, and IL‐1β, and thereby form a chronic neuroinflammatory microenvironment. This state not only directly impairs hippocampal neurons and synaptic function, leading to cognitive decline, but also drives the onset and progression of DE [258]. Single‐cell RNA sequencing studies have further uncovered that microglia in the hippocampus of diabetic mice undergo aberrant activation and exhibit proinflammation‐dominant heterogeneous subsets. These cells highly express inflammation‐ and immune‐related genes, alongside compromised oxidative stress defense capacity, which ultimately exacerbates hippocampal damage and impairs learning and memory function [259]. Notably, Th22 cells—potent cytokines capable of regulating multiple key cellular pathways in tissues—cross the BBB to infiltrate the hippocampus, where they secrete IL‐22. This cytokine facilitates the transition of homeostatic microglia to reactive microglia, thereby triggering inflammatory responses, worsening learning and memory impairments as well as cognitive deficits, and ultimately propelling and accelerating DE progression [260].

Accordingly, targeting neuroinflammatory modulation has emerged as a promising therapeutic strategy for DE. For instance, ablation of IL‐17A reduces hippocampal neuronal apoptosis and ameliorates DE‐associated cognitive deficits by attenuating systemic and central inflammatory responses, preserving BBB integrity, suppressing aberrant microglial activation, and reducing the accumulation of Alzheimer's disease‐related pathological proteins [261]. Current research on neuroinflammation in DE remains largely correlative, lacking precise understanding of key signaling pathways’ upstream triggers and downstream effects. Discrepancies between animal models and human disease further limit target specificity and clinical translation. Future studies should integrate cutting‐edge technologies like spatial multiomics to delineate cell‐specific regulatory networks in neuroinflammation and explore novel brain–peripheral immune interactions, ultimately bridging mechanistic discovery and clinical intervention.

#### 5.1.2 Neuroplasticity

In DE, neuroinflammation stands as one of the key mechanisms underlying impaired synaptic plasticity. Specifically, hyperglycemia stimulates the activation of the mTOR pathway in hippocampal neurons, which then serves as an upstream signal to drive NF‐κB pathway activation. This cascade enhances the release of proinflammatory cytokines while suppressing the expression of BDNF and critical synaptic proteins, ultimately resulting in structural synaptic damage and cognitive decline [262]. In recent years, further mechanistic studies have found that the expression of death‐associated protein kinase 1 is upregulated in DE, which significantly impairs hippocampal synaptic plasticity by inhibiting the expression of netrin‐1 [263]. Additionally, chronic hyperglycemia induces widespread abnormalities in the expression of synapse‐related genes—particularly in oligodendrocyte precursor cells—characterized by dysregulation of dendritic development, presynaptic assembly, and neuroactive ligand–receptor interactions, accompanied by perturbations in synaptic transmission, glutamatergic signaling, and long‐term depression [264]. Notably, another study demonstrated that PRG‐1 is involved in regulating hippocampal synaptic plasticity during diabetes‐induced cognitive impairment by modulating the nerve growth factor/BDNF/tropomyosin receptor kinase B (TrkB) signaling pathway [265].

Current research, while revealing multiple independent pathways underlying synaptic plasticity impairment in DE, has not yet elucidated the interconnections and hierarchical networks among these pathways. There is also a lack of integrated therapeutic strategies capable of simultaneously targeting multiple pathological mechanisms. Future studies should focus on mapping the interactions of multiple signaling pathways and exploring multitarget combination therapies centered on synaptic protection, thereby facilitating the translation of fundamental discoveries into clinical interventions.

#### 5.1.3 Mitochondria

In Type 2 diabetes, insulin resistance and hyperglycemia can trigger mitochondrial dysfunction, characterized by reduced energy production, excessive ROS generation, and impaired mitophagy and mitochondrial quality control mechanisms. Such dysfunction not only represents a key event in the pathogenesis of diabetes but also constitutes a molecular bridge linking Type 2 diabetes to neurodegenerative disorders such as Alzheimer's disease and Parkinson's disease [266]. In DE, mitochondrial function is further impaired, as evidenced by a reduction in mitochondrial membrane potential. Specifically, the upregulation of kinesin contributes to defective axonal transport, which in turn exacerbates the loss of membrane potential and mitochondrial dysfunction [267]. Mechanistically, diabetes upregulates the expression of cyclophilin D, which strengthens its interaction with the F_1_F_0_ ATP synthase and induces the abnormal opening of the mitochondrial permeability transition pore. This cascade ultimately results in synaptic dysfunction and cognitive decline [268].

Moreover, high glucose‐induced Lipin1 dysregulation disrupts mitochondrial‐associated endoplasmic reticulum membrane (MAM) homeostasis, leading to decreased membrane potential, exacerbated oxidative stress, and hyperactivation of mitophagy, which collectively impair neuronal energy metabolism and calcium homeostasis [269]. Notably, Lipin1 has also been shown to ameliorate cognitive function in DE by modulating calcium transfer at MAMs [270]. While current studies have pinpointed multiple mitochondria‐related pathways, they have yet to clarify their synergistic effects and hierarchical relationships in DE. Additionally, there remains a paucity of integrated intervention strategies capable of holistically improving mitochondrial function. Future research could further investigate the pathological mechanisms of mitochondria in DE, develop specific regulatory approaches targeting mitochondria, and facilitate the translational shift from mechanistic elucidation to clinical therapies focused on mitochondrial protection.

### 5.2 Treatment

#### 5.2.1 Natural Products

Multiple natural products have shown potential therapeutic value in DE, and their mechanisms of action involve multiple signaling pathways. Quercetin is a common flavonoid natural product that can exert neuroprotective effects in DE by regulating the SIRT1/NLRP3 signaling pathway [271]. Kuwanon G, a natural compound derived from mulberry, has been identified through network pharmacology studies as a potential therapeutic agent for DE, likely via modulation of the PI3K/Akt signaling pathway [272]. Dendrobine, an alkaloid isolated from Dendrobium officinale, ameliorates cognitive dysfunction in DE by activating the Nrf2/GPX4 axis to suppress ferroptosis [273]. Notoginsenoside R1, a key bioactive compound from Panax notoginseng, enhances neuronal glucose uptake in a murine model of diabetes‐associated Alzheimer's disease. This effect is mediated by the activation of the PPARγ/Akt/glucose transporter 4 (GLUT4) signaling pathway [274]. α‐Asarone is one of the major bioactive components of *Acori Tatarinowii Rhizoma*, and studies have shown that it ameliorates DE by enhancing mitophagy, suppressing apoptosis, and alleviating oxidative stress [275]. Reports also indicate that Resveratrol, a plant polyphenol, can regulate mitochondrial function via the phosphodiesterase‐4D‐dependent pathway to ameliorate cognitive impairment in db/db mice [276]. Salidroside is a natural product isolated from *Rhodiola rosea L*., and its mechanism of action in treating diabetic cognitive impairment involves activating the PI3K/AKT/glycogen synthase kinase 3β signaling pathway [277]. Gastrodin is one of the major components of *Gastrodia elata*. Studies have demonstrated that gastrodin can alleviate cognitive impairment in DE by inhibiting hippocampal endoplasmic reticulum stress and the activation of the NLRP3 inflammasome [278].

#### 5.2.2 Compounds

Not only is metformin widely used as a first‐line therapeutic agent for Type 2 diabetes mellitus due to its potent hypoglycemic effect, favorable safety profile, and cost effectiveness [279]. Beyond its well‐established role in regulating glucose metabolism, studies have found that it enhances autophagy via an AMPK‐dependent pathway, significantly attenuating tau pathology and improving cognitive function in db/db mice [280]. Further studies have also revealed that when metformin is combined with α‐lipoic acid, it synergistically activates the Nrf2/AMPK signaling pathway, offering a novel therapeutic strategy for improving cognitive impairment in T2DM‐related encephalopathy [281]. Beyond metformin, other compounds have also demonstrated potential in combating DE. Fasudil, a Rho kinase inhibitor, has effectively alleviated diabetes‐related cognitive decline in diabetic stroke model mice by inhibiting the NLRP3/caspase‐1/GSDMD signaling pathway [282]. Propionate, a SCFA commonly used as a preservative, can improve diabetic neurological dysfunction via the PI3K/Akt/endothelial nitric oxide synthase signaling pathway [283]. Additionally, sacubitril/valsartan plays a crucial role in ameliorating cognitive impairment observed in diabetic rats through antioxidant, anti‐inflammatory, and antiapoptotic effects [284]. H_2_S can regulate the SIRT1–mTOR/NF‐κB pathway to exert anti‐inflammatory effects in a high‐glucose environment, holding potential for the treatment of DE [285] (Table 4).

**TABLE 4:** Information on natural products and compounds in DE.

Natural product/compound	CAS No.	Molecular formula	Modeling method	Dose	Molecular mechanism	References
Quercetin	117‐39‐5	C_15_H_10_O_7_	−	35 and 70 mg/kg	Adjust SIRT1/NLRP3 pathway	[271]
Kuwanon G	75629‐19‐5	C_40_H_36_O_11_	−	20 mg/kg	Regulating the PI3K/Akt signaling pathway	[272]
Dendrobine	2115‐91‐5	C_16_H_25_NO_2_	In vivo: – In vitro: AGEs	In vivo: 10 and 20 mg/kg In vitro: 10 and 40 µM	Activate Nrf2/GPX4 axis to inhibit ferroptosis	[273]
Notoginsenoside R1	80418‐24‐2	C_47_H_80_O_18_	In vivo: – In vitro: Glucose (50 mM)	In vivo: 10, 20, 40, 80 mg/kg In vitro: 2.5, 5, 10, 20 µM	Activate the PPARγ/Akt/GLUT4 signaling pathway	[274]
α‐Asarone	2883‐98‐9	C_12_H_16_O_3_	In vivo: STZ In vitro: high‐glucose	In vivo: 30 and 60 mg/kg In vitro: 0.5, 1, 2, 4, 8 µg/mL	Activate mitochondrial autophagy, protect mitochondrial function, improve oxidative stress and cell apoptosis	[275]
Resveratrol	501‐36‐0	C_14_H_12_O_3_	In vivo: – In vitro: high‐glucose	In vivo: 10, 20, 40 mg/kg In vitro: 0.5, 1, 5 µmol/L	Regulating the PDE4D/PKA/Drp1 signaling pathway	[276]
Salidroside	10338‐51‐9	C_14_H_20_O_7_	STZ	100 mg/kg	Activate the PI3K/Akt/GSK‐3β signaling pathway	[277]
Gastrodin	62499‐27‐8	C_13_H_18_O_7_	−	70 and 140 mg/kg	Inhibition of hippocampal endoplasmic reticulum stress and activation of NLRP3 inflammasome	[278]
Metformin	657‐24‐9	C_4_H_11_N_5_	In vivo: – In vitro: high‐glucose	In vivo: 200 mg/kg In vitro: 3.2 mM	Enhance autophagy activity in an AMPK‐dependent manner	[280]
Fasudil	103745‐39‐7	C_14_H_17_N_3_O_2_S	High‐fat diet and STZ	10 mg/kg	Inhibition of NLRP3/caspase‐1/GSDMD signaling pathway	[282]
Propionate	72‐03‐7	C_3_H_5_O_2_	STZ	37.5, 75, and 150 mg/kg	Regulating the PI3K/Akt/eNOS signaling pathway	[283]
Hydrogen sulfide	7783‐06‐4	H_2_S	High‐glucose	250 µM	Regulating the SIRT1–mTOR/NF‐κB signaling pathway	[285]

*Abbreviations*: Akt, protein kinase B; DE, diabetic encephalopathy; GPX4, glutathione peroxidase 4; GSK‐3β, glycogen synthase kinase 3β; mTOR, mammalian target of rapamycin; NF‐κB, nuclear factor kappa B; NLRP3, NOD‐like receptor protein 3; Nrf2, nuclear factor‐E2‐related factor 2; PDE4D, phosphodiesterase‐4D; PI3K, phosphatidylinositol 3‐kinase; PPARγ, peroxisome proliferator‐activated receptor γ.

Although current studies have indicated the therapeutic potential of certain natural compounds and compounds in DE, several key limitations persist. The evidence remains largely preclinical, lacking systematic clinical validation. Research focus has been predominantly on neuronal protection, with insufficient attention to multicellular interactions within the neurovascular unit or system‐level mechanisms such as cross‐organ communication via the MGB axis. Furthermore, combination therapies involving multitarget natural agents are underexplored. Future efforts should prioritize the development of multitarget, multimodal treatment systems, supported by advanced tools such as spatial transcriptomics, brain organoids, and artificial intelligence, to decipher complex regulatory networks and foster personalized, integrated pharmacological and nonpharmacological interventions.

## 6 Uremic Encephalopathy

Uremic encephalopathy is a CNS complication in patients with renal failure, primarily induced by metabolic disturbances. Its pathogenesis is complex and may involve multiple factors, including retention of uremic toxins, BBB dysfunction, and activation of inflammatory responses. The disease lacks specific clinical manifestations, laboratory biomarkers, or imaging characteristics, and its diagnosis is often retrospectively established only after neurological symptoms improve following dialysis or kidney transplantation [286, 287]. The current lack of targeted therapeutic strategies, largely attributable to an incomplete understanding of its core pathophysiological mechanisms, remains a major bottleneck hindering breakthroughs in clinical management.

### 6.1 Causes and Pathological Mechanisms

The kidneys remove metabolic wastes from the body, including creatinine, urea, and uric acid. When these metabolic wastes are not adequately removed by the kidneys, the accumulation of uremic toxins affects the equilibrium of the body's internal environment, resulting in damage to tissues and organs throughout the body [288]. Notably, uremic toxins can cross the BBB and enter the brain. Under physiological conditions, organic anion transporter 3 (OAT3) is responsible for transporting protein‐bound uremic toxins from brain tissue back to the bloodstream; however, in patients with chronic kidney disease, the function of this transporter is impaired, leading to the accumulation of uremic toxins in the brain. These retained metabolic wastes then interact with brain cells such as neurons and astrocytes, ultimately impairing the cerebrovascular structure and intracerebral substance transport pathways in patients with renal insufficiency [289, 290, 291].

Uremia exacerbates oxidative stress and neuroinflammation in the brain, while concurrently suppressing the Nrf2‐mediated antioxidant defense pathway and downregulating key BBB proteins [292]. Further corroborating this, recent studies have demonstrated that hippuric acid, a uremic toxin accumulated in both the blood and brain following renal injury, promotes ROS overproduction, inhibits the Nrf2/HO‐1 signaling pathway, and abnormally upregulates the expression and activity of cytochrome P450 enzyme CYP2D4, thereby contributing to the pathogenesis of uremic encephalopathy [293].

Neuroinflammation also serves as a critical mediator in the pathogenesis of uremic encephalopathy. For instance, indoxyl sulfate activates both peripheral macrophages and cerebral microglia, promoting the release of TNF‐α. This cytokine subsequently inhibits OAT3 function, creating a vicious cycle of impaired toxin clearance, while also downregulating the expression of synaptic proteins and microtubule‐associated protein 2, ultimately leading to cognitive and behavioral deficits [294]. In fact, indoxyl sulfate has been identified as a key uremic toxin responsible for neuropsychiatric symptoms in patients with chronic kidney disease [295]. Similarly, in a unilateral nephrectomy mouse model, p‐cresol sulfate activates microglia, significantly elevates levels of proinflammatory cytokines such as IL‐1β in the prefrontal cortex and serum, and triggers neuroinflammatory signaling pathways, thereby inducing cognitive impairments and behavioral alterations [296].

Reports have also shown that serum from patients with chronic kidney disease can induce an inflammatory response in astrocytes, with the severity being proportional to the concentration of indoxyl sulfate, while the removal of indoxyl sulfate using the oral adsorbent AST‐120 significantly alleviates this inflammatory response. Further mechanistic studies have demonstrated that indoxyl sulfate can exacerbate inflammatory responses and oxidative stress in CNS cells by activating the NF‐κB and aryl hydrocarbon receptor signaling pathways, ultimately leading to neuronal death [297]. Additionally, chronic kidney disease can disrupt the BBB, allowing uremic toxins to enter the brain and cause dysregulation of calcium homeostasis in microglia, which in turn promotes increased potassium efflux via the calcium‐dependent potassium channel K_Ca_3.1, activates the p38–MAPK pathway, and facilitates the maturation and release of IL‐1β; the released IL‐1β then acts on the IL‐1R1 receptor on neurons, leading to reduced potassium turnover and functional abnormalities in neurons, and ultimately triggering cognitive impairment [298].

Uremic toxins act through intertwined multiple mechanisms to collectively contribute to the pathological process of chronic kidney disease‐related brain damage, but whether there are synergistic or antagonistic effects between different uremic toxins and their dynamic influences on specific brain regions and cell types at different disease stages remain unclear. In addition, mechanistic studies show a fragmented tendency, failing to fully reveal the causal relationships and chronological order between different pathological processes such as oxidative stress, neuroinflammation, electrolyte disorders, and decreased synaptic plasticity, which makes it difficult to construct a comprehensive map of the disease cascade reactions. Finally, there is currently a lack of ideal animal models that can effectively simulate the progressive characteristics of human chronic kidney disease, and this limits the in‐depth study of the mechanisms throughout the entire disease course and the accurate evaluation of clinically translational intervention strategies.

### 6.2 Treatment

ω‐3 Polyunsaturated fatty acids (ω‐3 PUFAs), a class of dietary essential PUFAs, play a crucial role in maintaining body health. Studies have shown that they can enhance the survival of nerve cells and inhibit apoptosis by activating the PI3K–Akt signaling pathway, thereby directly alleviating the damage of uremic toxins to hippocampal neurons. In addition, ω‐3 PUFAs can also alleviate neuroinflammation and oxidative stress by inhibiting the JNK/MAPK signaling pathway, further restoring the integrity of the BBB and ultimately improving cognitive function and synaptic plasticity [299, 300]. Meanwhile, metformin combined with low‐dose radiation exerts a synergistic effect in renal function protection and alleviation of brain tissue damage by activating the AMPK signaling pathway, enhancing autophagy, and inhibiting oxidative stress, inflammatory responses, and cell apoptosis [301].

Of note, dimethyl fumarate (a small‐molecule drug) not only ameliorates acute kidney injury and renal function by activating the Nrf2 antioxidant pathway and inhibiting inflammatory signals such as NF‐κB in the kidneys, but also suppresses the IL‐6/STAT3 and NF‐κB neuroinflammatory pathways in the brain, alleviates abnormal astrocyte activation and neurodegeneration, and achieves dual renal and neural protection [302]. Similarly, nobiletin, a natural flavonoid compound with potential neuroprotective effects, has also been shown to alleviate uremia‐induced brain damage and neuronal apoptosis, as well as improve renal function, by activating the PI3K/Akt signaling pathway [303]. On the other hand, in a rat model of renal ischemia–reperfusion, acute kidney injury was found to increase oxidative stress levels and nitric oxide metabolites in the hippocampus, accompanied by impairments in spatial memory and passive avoidance memory, as well as compromised hippocampal neuroplasticity. However, losartan treatment not only improves renal function parameters but also significantly restores cognitive behavioral performance and synaptic plasticity by reducing hippocampal oxidative stress [304] (Table 5).

**TABLE 5:** Information on natural products and compounds in uremic encephalopathy.

Natural product/compound	CAS No.	Molecular formula	Modeling method	Dose	Molecular mechanism	References
Omega‐3 polyunsaturated fatty acid	−	−	Ischemia–reperfusion	4 g/kg	Regulating the PI3K/Akt signaling pathway	[299]
			Ischemia–reperfusion and omnihexol	4 g/kg	Inhibiting the JNK/MAPK signaling pathway, alleviating neuroinflammation and oxidative stress, and thereby repairing BBB integrity	[300]
Dimethyl fumarate	624‐49‐7	C_6_H_8_O_4_	Ischemia–reperfusion	15 and 25 mg/kg	Has a dual protective effect on the kidneys and nerves	[302]
Nobiletin	478‐01‐3	C_21_H_22_O_8_	Cisplatin	In vivo: 100 mg/kg In vitro: 25, 50, 100 µM	Activate the PI3K/Akt signaling pathway	[303]
Losartan	114798‐26‐4	C_22_H_23_ClN_6_O	Acute kidney injury	10 mg/kg	Reduce hippocampal oxidative stress	[304]

*Abbreviations*: Akt, protein kinase B; JNK, Jun N‐terminal kinase; MAPK, mitogen‐activated protein kinases; PI3K, phosphatidylinositol 3‐kinase.

Collectively, the aforementioned studies reveal that intervention strategies targeting different molecular targets all demonstrate potential therapeutic value in alleviating uremia‐related brain damage; however, most current evidence is limited to preclinical studies, lacking data on human safety and efficacy, and most interventions are single‐target, failing to fully reflect the complex pathological network involving crosstalk between multiple pathways in uremic encephalopathy. The future development trend should focus on advancing candidate compounds with well‐defined mechanisms into early‐phase clinical trials, exploring multitarget combination therapy strategies, and developing personalized, stage‐adapted comprehensive intervention regimens—all of which will be the key direction for achieving effective clinical management of uremic encephalopathy.

## 7 Toxic Encephalopathies

Toxic encephalopathy is a CNS dysfunction caused by neurotoxins, with common etiologies including heavy metals, among others. Its clinical manifestations are diverse, which can present as acute or chronic cognitive impairment, altered consciousness, emotional disturbances, and motor ataxia. Diagnosis is mainly based on a detailed toxin exposure history and clinical manifestations, supplemented by neuroimaging, neurophysiology, and laboratory tests for specific toxins [305]. Currently, the specific pathophysiological mechanisms of this disease have not been fully elucidated, and it still faces numerous challenges in terms of diagnosis and treatment.

### 7.1 Causes and Pathological Mechanisms

#### 7.1.1 Manganese

Given that manganese is widely used in fields such as nonferrous metals, chemical engineering, agriculture, and medicine, large amounts of this element are released into the ecological environment, leading to its accumulation year by year and posing a potential threat to human health and the health of other organisms. Epidemiological studies have confirmed that long‐term exposure to environments where manganese concentrations exceed 0.2 mg/m^3^ in air or 300 µg/L in drinking water significantly increases the risk of developing neurological diseases [306, 307]. Mechanistic studies have revealed that manganese exposure activates the cGAS/STING pathway, mediates the production of ROS, induces apoptosis and ferroptosis, and triggers taupathy mediated by autophagolysosomal pathway dysfunction, thereby causing neuronal damage [308, 309]. Meanwhile, manganese exposure can also induce Parkinson‐like symptoms through lysosomal dysfunction mediated by the Serpina3n‐transcription factor EB (TFEB)‐v/p‐ATPase signaling axis [310]. Additionally, in manganese‐exposed mice, excessive mitochondrial stress response causes neuronal damage via sustained phosphorylation of eukaryotic translation initiation factor 2α (eIF2α) [311]. Manganese can also stimulate ferroptosis through ferritinophagy mediated by nuclear receptor coactivator 4 (NCOA4), inducing neurotoxicity in mice and hippocampal HT22 cells [312].

Reports have also shown that manganese primarily initiates its neurotoxicity by inducing mitochondrial dysfunction: it accumulates in mitochondria, inhibits the electron transport chain, reduces ATP synthesis, generates large amounts of ROS to trigger oxidative stress, disrupts intracellular calcium homeostasis, and inhibits normal calcium signaling—all of which collectively impair neuronal excitability and synaptic function. Meanwhile, manganese can also induce caspase‐mediated apoptosis, ultimately leading to impaired neuronal function [313]. Overall, manganese is not only involved in the pathogenesis and progression of HE but also induces neurotoxicity through multiple pathways, including oxidative stress, ferroptosis, dysregulation of the autophagy–lysosome system, mitochondrial dysfunction, and calcium homeostasis imbalance. These findings not only deepen the understanding of the pathophysiological processes of manganese toxicity but also provide a theoretical basis for identifying therapeutic targets.

#### 7.1.2 Lead

Lead is a pervasive environmental pollutant that continues to pose a significant threat to public health worldwide. With economic and technological advancement, the use of lead has become increasingly widespread. Notably, growing demands for electricity and universal communication have driven its extensive application in battery manufacturing and computer circuit boards. This has led to rising lead levels in soil, water, and the atmosphere, resulting in substantial human exposure through both the digestive and respiratory tracts [314]. In studies on lead‐induced toxic encephalopathy, multiple molecular and cellular pathways have been elucidated. The P62/Nrf2/Keap1 signaling pathway has been shown to mediate lead‐induced neuronal dysfunction [315]. Furthermore, disrupted neurotransmitter‐related communication between microglia and neurons represents a key mechanism underlying lead neurotoxicity [316]. Moreover, even low‐level lead exposure can perturb striatal dopaminergic signaling and induce behavioral deficits, providing mechanistic insight into the link between lead exposure and disorders such as attention deficit hyperactivity disorder and behavioral abnormalities [317].

At the mitochondrial level, lead exposure significantly impairs mitochondrial number and morphology in SH‐SY5Y neuroblastoma cells by disrupting mitochondrial dynamics [318]. Notably, inhaled lead nanoparticles can enter the brain via the olfactory pathway and the impaired BBB, and accumulate in key brain regions such as the hippocampus and cortex. The core neurotoxic mechanism of lead lies in lead ions disrupting intracellular calcium homeostasis, thereby destabilizing cytoskeletal structure, inducing Alzheimer's disease‐like Tau protein pathological changes, and causing structural damage such as neuronal necrosis and nuclear vacuolation [319].

As an environmental pollutant, lead enters the human body through multiple pathways and accumulates in the brain, where it triggers neurotoxicity via mitochondrial dysfunction, disrupted neuronal communication, and oxidative stress, ultimately causing irreversible neurodegeneration. However, research on chronic low‐dose exposure remains limited, lacking both adequate neurobehavioral impact assessment and sensitive early biomarkers. Furthermore, despite growing mechanistic knowledge, effective clinical strategies to specifically protect neurons or block lead's toxic progression are still scarce, underscoring an urgent need to advance translational research in neuroprotective and detoxification approaches.

#### 7.1.3 Cadmium

Cadmium is a known nonessential hazardous heavy metal and human carcinogen that is widely prevalent in the environment. It may enter the human body by inhalation, ingestion, or cutaneous contact, and has a biological half‐life of 25–30 years, causing increasing buildup and long‐term harm to cells and organs [320]. Accumulating evidence indicates that cadmium is a potential risk factor for CNS disorders. For example, childhood cadmium exposure has been shown to exert significant adverse effects on cognitive ability in boys [321]. Cadmium can induce brain damage via the MGB axis. Specifically, cadmium first disrupts the homeostasis of gut microbiota and SCFAs, impairs intestinal barrier function, and promotes the entry of inflammatory substances into the circulation, which further damages the BBB; this thereby activates neuroinflammatory responses in the hippocampus while downregulating the expression of key genes associated with learning, memory, and synaptic plasticity [322]. Additionally, maternal exposure to the environmental pollutant cadmium during pregnancy and lactation leads to persistent learning and memory impairments in male offspring, whereas female offspring remain unaffected. The core mechanism lies in that cadmium exposure specifically upregulates phospholipase C (PLC) β4 protein in the hippocampus of male offspring; this protein competes with PLCγ1 for binding to phosphatidylinositol 4,5‐bisphosphate, thereby inhibiting the critical neuroprotective PLCγ1/cAMP response element‐binding protein (CREB)/BDNF signaling pathway and ultimately resulting in neuronal damage and cognitive deficits [323].

On the other hand, cadmium disrupts sphingolipid metabolism in the midbrain, leading to increased levels of lipids such as proinflammatory ceramides, thereby triggering neuroinflammation [324]. Long‐term low‐dose cadmium exposure impairs cognitive function by activating mitochondrial fission and dysfunction mediated by the long noncoding RNA Gm10532 (lnc‐Gm10532)/m6A/fission 1 (FIS1) axis [325]. Of note, neurogenesis, which is a key process in the adult brain involving the generation and integration of new neurons into existing neural circuits, plays a crucial role in cognitive function; whereas cadmium exposure can impair adult hippocampal neurogenesis by disrupting store‐operated calcium entry [326]. Taken together, cadmium, as a persistently existing neurotoxic heavy metal in the environment, can impair the CNS through multiple mechanisms including the MGB axis, epigenetic regulation, mitochondrial dysfunction, and neurogenesis inhibition. Its toxic effects exhibit gender‐specificity and developmental stage‐specific differences, with particularly significant impacts on early cognitive function and male offspring. These research findings systematically reveal the key mechanisms underlying cadmium neurotoxicity, which not only deepen the understanding of relevant pathophysiological processes but also provide an important theoretical basis for the development of targeted intervention strategies in the future.

#### 7.1.4 Arsenic

Arsenic is a toxic metalloid widely distributed in the environment, and its presence in soil, food, and water exposes humans to arsenic inevitably [327]. Early life arsenic exposure leads to neurodevelopmental disorders in offspring; studies have shown that arsenic primarily induces histone H3K9 methylation, thereby causing PANoptosis and neurodevelopmental disorders in offspring [328]. Furthermore, early life arsenic exposure triggers dynamic shortening of telomere length in the hippocampus. This change impairs the proliferation, differentiation, and migration abilities of neural stem cells, disrupts the dendritic complexity of neurons and the ultrastructure of synapses, and downregulates the expression of key neurodevelopmental genes, ultimately resulting in cognitive impairments in spatial learning and memory in offspring [329].

In terms of neuroinflammatory mechanisms, developmental arsenic exposure activates the NLRP3 inflammasome pathway in microglia, promotes the release of proinflammatory factors such as IL‐1β, triggers neuroinflammation in the hippocampus, and thereby reduces the levels of synaptic proteins PSD95 and synaptophysin, causes neuronal damage, and ultimately results in learning and memory deficits [330]. Of note, long‐term arsenic exposure can induce Parkinson's disease‐like neurodegenerative changes; the underlying mechanism involves inhibiting the activity of mitochondrial Complex II and IV in key brain regions (particularly the substantia nigra), leading to energy metabolism disorders and activating inflammatory pathways in astrocytes and neurons [331].

Meanwhile, long‐term arsenic exposure also causes Alzheimer's disease‐like pathological changes in mice, characterized by decreased cognitive function and anxiety‐like behaviors, accompanied by a significant increase in acetylcholinesterase activity and elevated expression of neuroinflammatory markers in the cerebral cortex and hippocampus [332]. In a study combining RNA sequencing and bioinformatics analysis, it was found that arsenic disrupts a sophisticated posttranscriptional regulatory network composed of mRNA, lncRNA, circRNA, and miRNA, thereby potentially causing neuronal damage [333]. These studies systematically reveal the multimechanism and multistage characteristics of arsenic neurotoxicity, providing an important scientific basis for in‐depth understanding of its pathological processes.

#### 7.1.5 Aluminum

Aluminum is a ubiquitous environmental and industrial pollutant that enters the human body via inhalation, ingestion, and dermal routes. Studies have shown that aluminum‐induced neurotoxicity involves activating the NLRP3 inflammasome‐mediated pyroptosis pathway, thereby triggering neuroinflammation and cell death [334]. Further mechanistic studies have revealed that aluminum exposure upregulates the expression of miR‐98‐5p, which targets and inhibits insulin‐like growth factor 2; this in turn inhibits its downstream JAK2/STAT3 signaling pathway, ultimately leading to neuronal apoptosis, a decreased number of hippocampal neurons, and impaired learning and memory abilities in rats [335]. Additionally, reports have found that aluminum‐induced neurotoxicity involves modifications to m6A RNA [336].

Of note, alumina nanoparticles are widely used in fields such as cosmetics, industry, medicine, and vaccines; however, these particles are released into soil, air, and water during their production, transportation, use, and disposal, significantly increasing occupational and environmental exposure risks. For instance, after acute exposure, alumina nanoparticles cause damage through initial inflammation and oxidative stress in a size‐dependent manner; in contrast, long‐term exposure leads to time‐dependent damage via progressively exacerbated oxidative stress and neuronal death [337]. Additionally, alterations in epigenetic markers such as hippocampal histone acetylation and methylation are among the key mechanisms underlying aluminum‐induced learning and memory impairments in rats [338]. Current research, while identifying multiple mechanisms in aluminum neurotoxicity, lacks systematic analysis of their synergies and hierarchy—especially regarding progressive damage from long‐term, low‐dose exposure. Future studies should leverage multiomics and other advanced technologies to construct a global molecular network of aluminum toxicity, with the ultimate goal of translating mechanistic insights into neuroprotective strategies for clinical intervention.

#### 7.1.6 Plastic

Microplastics and nanoplastics, due to their global distribution and environmental persistence, result in the inevitable continuous exposure of humans and animals to them. Existing studies have shown that these plastic particles can be absorbed by aquatic organisms and mammals, cross physiological barriers to enter the brain, and pose potential hazards to the nervous system [339]. Mechanistic studies have revealed that polystyrene nanoplastics (PS‐NPs) may induce neuronal cuproptosis via oxidative stress‐mediated activation of the ERK/MAPK pathway, thereby causing cognitive impairment in mice [340]. From a neuroimmunological perspective, further research has found that PS‐NPs activate the NF‐κB signaling pathway and promote the release of TNF‐α and IL‐1β, thus driving neuroinflammatory responses [341]. Notably, early life exposure to PS‐NPs can induce transgenerational neurotoxicity. When maternal mice are exposed to environmentally relevant doses of PS‐NPs during pregnancy and lactation, it leads to gut microbiota dysbiosis in offspring, which thereby disrupts the intestinal barrier, allows LPS to enter the bloodstream, triggers cerebral neuroinflammation and brain tissue damage, and interferes with the metabolism and signal transduction of key neurotransmitters such as dopamine and serotonin [342]. In another study on early life PS‐NP exposure, it was found that PS‐NPs disrupt the gut microbiota and reduce the level of its metabolite trehalose; this subsequently causes lysosomal and proteasomal dysfunction in the brain, impairs protein homeostasis, and ultimately induces neuronal damage and behavioral abnormalities [343]. In a zebrafish model, early life exposure to PS microplastics was found to induce behavioral manifestations similar to attention deficit hyperactivity disorder, including hyperactivity and impulsivity. The underlying mechanism is that microplastics disrupt the normal development of dopaminergic neurons, leading to an abnormal increase in the number of such neurons in the brain and causing dysregulation of gene expression in dopamine‐related signaling pathways [344].

Furthermore, coexposure studies indicate that ozone and PS‐NPs synergistically induce neurobehavioral deficits in mice. Their combined effect disrupts BBB integrity, enhances oxidative stress, activates microglia, and triggers neuroinflammation, ultimately leading to neuronal pyroptosis via the p38–MAPK signaling pathway, which exacerbates cognitive impairment and anxiety‐like behavior [345]. In a separate study, polypropylene microplastics were found to act as carriers that facilitate the accumulation of oxytetracycline in the brains and intestines of fish, aggravating histopathological damage. This exposure also altered the gut microbiota composition, increased the abundance of pathogenic bacteria, significantly reduced SCFA levels, and disrupted neurotransmitter balance, collectively contributing to neurotoxicity [346]. Current studies have demonstrated that micro‐NPs cause neurotoxicity via pathways including the MGB axis, oxidative stress, and neuroinflammation. However, systematic comparisons are still lacking regarding polymer‐specific toxicity, cumulative effects of chronic low‐dose exposure, and life stage‐specific susceptibility. Future work should develop animal models and risk assessment frameworks for real‐world mixed exposures, while also advancing early neurotoxicity biomarkers and targeted intervention strategies.

### 7.2 Treatment

#### 7.2.1 Natural Products

In studies on toxic encephalopathy, a certain number of studies have reported the neuroprotective effects of natural products. For example, quercetin—a common flavonoid natural product—has been reported to reduce neuronal and non‐neuronal apoptosis by enhancing antioxidant capacity, thereby alleviating the adverse effects of sodium fluoride on the medial prefrontal cortex of adult rats [347]. Naringin can exert a mitigating effect on neurodamage induced by cadmium and a high‐fat diet by reducing lipid peroxidation, enhancing the activity of endogenous antioxidant enzymes, improving mitochondrial function and integrity, restoring acetylcholinesterase activity, and significantly reducing neuroinflammation [348]. Diazinon is a commonly used organophosphorus insecticide, widely employed in pest control particularly in developing countries. However, humans and other nontarget organisms still sustain toxic effects upon exposure to this pesticide, which remains an important concern in the field of public health. In diazinon‐induced neurodamage, epigallocatechin‐3‐gallate alleviates diazinon‐induced neurotoxicity by inhibiting the expression of proinflammatory genes and enhancing antioxidant pathways [349]. Cadmium is a ubiquitous environmental pollutant and has attracted significant attention due to its neurotoxicity. Therefore, identifying effective intervention measures for cadmium‐induced neurodamage is crucial. The flavonoid compound Daidzein exerts a neuroprotective effect on the cerebral cortex in a cadmium‐induced neurotoxicity model in rats by inhibiting mitochondrial signaling pathway‐mediated apoptosis and PINK1/Parkin‐mediated mitophagy [350]. Additionally, luteolin can also exert a therapeutic effect on cadmium‐induced neurotoxicity; its mechanism of action involves inhibiting the activity of neuroinflammatory signaling pathways and upregulating the BDNF signaling pathway to promote neuronal survival [351].

Tomatoes and other red fruits and vegetables are rich sources of lycopene, a naturally occurring carotenoid pigment. By regulating the MGB axis, preventing neuroinflammation, and reestablishing intestinal homeostasis, lycopene can offer defense against Di(2‐ethylhexyl) phthalate‐induced neurotoxicity [352]. The prevalence of methamphetamine abuse has increased significantly in many parts of the world; it causes oxidative neurotoxicity by activating TRPV1‐dependent calcium influx and inducing oxidative stress and apoptosis. In contrast, cannabidiol inhibits methamphetamine‐induced oxidative neurotoxicity by modulating TRPV1 receptors [353]. Betaine is a type of trimethylglycine that mediates neuroprotective effects against sodium fluoride‐induced cerebellar damage through its antioxidant, anti‐inflammatory, and antiapoptotic activities; this effect is driven by its molecular interaction with Keap1‐Nrf2 [354].

Syringic acid is widely present in plants and fruits in nature, and it can exert neuroprotective effects in cadmium‐induced neurotoxicity by activating the Nrf2/HO‐1/SIRT1 signaling axis, inhibiting signal transduction of the TLR4/NF‐κB/JNK pathway, and upregulating the PI3K/Akt/mTOR pathway [355]. Similarly, β‐caryophyllene can also exert neuroprotective effects in cadmium‐induced neurotoxicity, with its mechanism of action involving the regulation of oxidative stress, inflammation, apoptosis, and autophagy [356]. The common metallic poison that may readily enter the brain and have serious pathological consequences is aluminum. However, by blocking the signaling molecules STAT3 and NF‐κB, eugenol reduces oxidative stress, neuronal damage, and reactive astrogliosis in rats poisoned with aluminum [357]. These studies systematically reveal that natural products combat neurodamage induced by different environmental toxins through multitarget and multipathway mechanisms, providing an important scientific basis for the development of neuroprotective strategies.

#### 7.2.2 Compounds

Acrylamide, a common neurotoxic compound, is widely present in tobacco and heat‐processed carbohydrate‐rich foods, and chronic exposure to it has become a non‐negligible public health concern. Studies have shown that melatonin can effectively mitigate Acrylamide‐induced neurotoxicity by alleviating endoplasmic reticulum stress, inhibiting inflammatory responses and apoptosis, with its mechanism involving reducing oxidative stress levels, restoring antioxidant enzyme activity, and decreasing the expression of proinflammatory cytokines [358]. Pretreatment with melatonin can also restore the synaptic morphological plasticity of hippocampal and cortical neurons, particularly in terms of spine density, by increasing SIRT1 expression, alleviate memory impairment induced by BDE‐209, and thereby provide a potential neuroprotective intervention [359].

**FIGURE 4:**
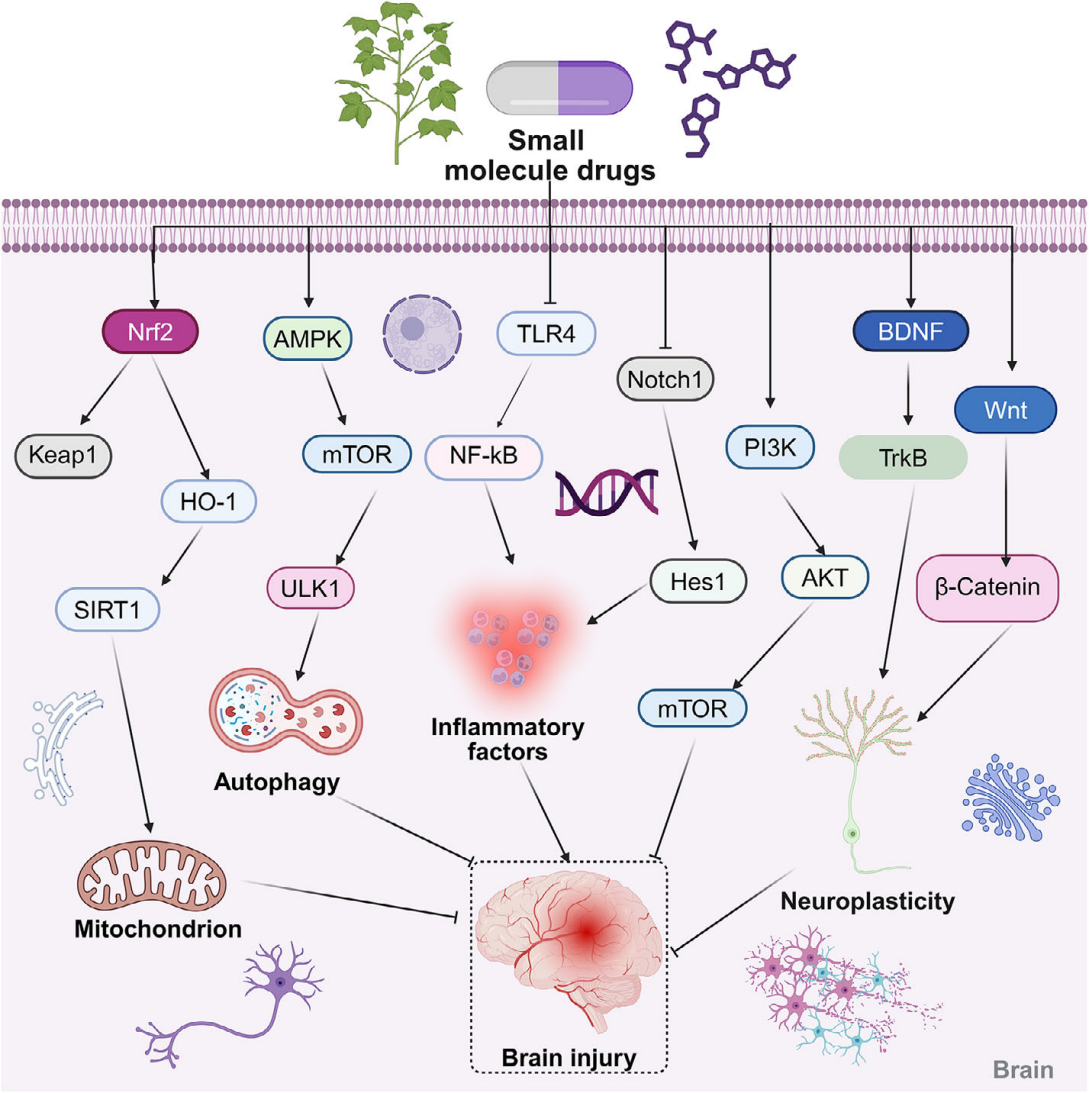
The molecular mechanism of small molecule drugs in toxic encephalopathy. *Abbreviations*: Nrf2, nuclear factor‐E2‐related factor 2; HO‐1, heme oxygenase‐1; SIRT1, sirtuin 1; AMPK, AMP‐activated protein kinase; mTOR, mammalian target of rapamycin; TLR4, Toll‐like receptor 4; NF‐κB, nuclear factor kappa B; PI3K, phosphatidylinositol 3‐kinase; AKT, protein kinase B; BDNF, brain‐derived neurotrophic factor; TrkB, tropomyosin receptor kinase B.

Besides melatonin, various compounds also exhibit protective potential in combating neurodamage induced by other environmental toxins. Metformin can significantly reduce oxidative stress, regulate key signaling pathways including Wnt/β‐catenin, and protect against aluminum‐induced neurodamage [360]. Rapamycin alleviates the neurotoxicity induced by fluoride and aluminum by activating autophagy in neurons and cells via the AMPK signaling pathway [361]. In terms of combined intervention, the combined application of CoQ10 and curcumin may alleviate memory loss induced by AlCl_3_ intoxication by restoring abnormal acetylcholinesterase activity, enhancing antioxidant defense, and rescuing detrimental neuronal damage [362] (Figure 4 and Table 6).

**TABLE 6:** Information on natural products and compounds in toxic encephalopathies.

Natural product/compound	CAS No.	Molecular formula	Modeling method	Dose	Molecular mechanism	References
Quercetin	117‐39‐5	C_15_H_10_O_7_	Sodium fluoride	25 mg/kg	Enhance antioxidant capacity to reduce neuronal and non‐neuronal apoptosis	[347]
Naringin	10236‐47‐2	C_27_H_32_O_14_	Cadmium and high‐fat diets	50 or 100 mg/kg	Reduced the synergistic neurotoxic effect of cadmium and HFD	[348]
Epigallocatechin‐3‐gallate	−	−	Diazinon	50 and 100 mg/kg	Inhibit oxidative stress and neuroinflammation	[349]
Daidzein	486‐66‐8	C_15_H_10_O_4_	Cadmium	100 mg/kg	Regulating mitochondrial function	[350]
Luteolin	491‐70‐3	C_15_H_10_O_6_	Cadmium	50 mg/kg	Inhibition of Notch1/Hes1 inflammatory signaling pathway and enhancement of BDNF signaling pathway activity	[351]
Lycopene	502‐65‐8	C_40_H_56_	Di(2‐ethylhexyl) phthalate	2.5, 5 and 10 mg/kg	Regulating MGB axis, inhibiting neuroinflammation and restoring intestinal homeostasis	[352]
Cannabidiol	13956‐29‐1	C_21_H_30_O_2_	Methamphetamine	In vivo: 40 and 80 mg/kg In vitro: 0.1, 1, 10 µM	Adjust TRPV1	[353]
Betaine	107‐43‐7	C_5_H_11_NO_2_	Sodium fluoride	50 and 100 mg/kg	Regulating the molecular interaction between Keap1 and Nrf2	[354]
Syringic acid	530‐57‐4	C_9_H_10_O_5_	Cadmium	50 and 100 mg/kg	Activate the Nrf2/HO‐1/SIRT1 signaling axis, inhibit the signal transduction of the TLR4/NF‐κB/JNK pathway, and upregulate the PI3K/Akt/mTOR signaling pathway	[355]
β‐caryophyllene	87‐44‐5	C_15_H_24_	Cadmium	200 and 400 mg/kg	Reduce oxidative stress, inflammation, apoptosis, and autophagy caused by cadmium‐induced neurotoxicity	[356]
Eugenol	97‐53‐0	C_10_H_12_O_2_	AlCl_3_	200 mg/kg	Inhibition of signal molecules STAT3 and NF‐κB	[357]
Melatonin	8041‐44‐9	C_13_H_16_N_2_O_2_	Acrylamide	10 and 20 mg/kg	Reduce oxidative stress levels, restore antioxidant enzyme activity, and decrease the expression of proinflammatory cytokines	[358]
			BDE‐209	10 mg/kg	Upregulation of SIRT1 expression restores synaptic plasticity, especially spike density, in hippocampal and cortical neurons	[359]
Metformin	657‐24‐9	C_4_H_11_N_5_	Aluminum	0.25 µM	Regulating key signaling pathways including Wnt/β‐catenin to protect against neuronal damage caused by aluminum	[360]
Rapamycin	53123‐88‐9	C_51_H_79_NO_13_	NaF and AlCl_3_	In vivo: 5 mg/kg In vitro: 20 µmol/L	Activate autophagy in neurons and cells	[361]

*Abbreviations*: Akt, protein kinase B; BDNF, brain‐derived neurotrophic factor; HO‐1, heme oxygenase‐1; MGB, microbiota–gut–brain; mTOR, mammalian target of rapamycin; NF‐κB, nuclear factor kappa B; Nrf2, nuclear factor‐E2‐related factor 2; PI3K, phosphatidylinositol 3‐kinase; TLR4, Toll‐like receptor 4; TRPV1, transient receptor potential vanilloid 1.

Current research has revealed the therapeutic potential of various natural products and compounds against toxic encephalopathy, yet most studies remain confined to models examining single compounds and individual exposure factors. There is a notable lack of systematic investigation under coexposure scenarios involving multiple pollutants, and the synergistic or antagonistic mechanisms among different protective agents remain unclear. Future studies should focus on constructing an integrated neuroprotective network map encompassing multiple targets and pathways, advance the development of combined intervention strategies based on real‐world exposure patterns, and strengthen translational research from animal models to clinical applications. These efforts will provide new avenues for the precise prevention and control of neurotoxicity.

## 8 Limitations and Challenges

The pathophysiological mechanisms of encephalopathies such as SAE, HE, HIE, DE, uremic encephalopathy, and toxic encephalopathy have not been fully elucidated. This knowledge gap has hindered breakthroughs in targeted therapeutic strategies. By systematically reviewing relevant literature in recent years, this article aims to in‐depth analyze the etiologies and pathogenesis of the aforementioned encephalopathies and review the current research progress in treatment with compounds and natural products. Notably, natural products exhibit unique advantages in encephalopathy treatment, including synergy of multiple components, multitarget intervention, and potential for individualized treatment, which highly align with the development trend of precision medicine. How to effectively leverage these advantages of natural products to overcome the limitation of traditional Western medicine with single targets, promote their application in multitargeted treatment of encephalopathies and other conditions, and thereby achieve synergistic efficacy comparable to the “cocktail therapy” in modern medicine, has become a key direction of current research and a hot topic of public concern.

However, this field still faces numerous challenges. Currently, most mechanistic studies on small‐molecule drugs remain in the preclinical stage, lacking support from high‐quality randomized double‐blind clinical trials, and their actual efficacy in improving the pathological symptoms of encephalopathy in patients as well as their mechanisms of action remain to be verified. On the other hand, the occurrence and development of encephalopathy are closely related to CNS damage, but many natural products have issues such as poor stability, low solubility, and difficulty in crossing the BBB. Moreover, most current studies have not clarified their distribution in brain tissue, and whether they can accurately target the CNS remains to be elucidated. Furthermore, natural resources derived from traditional medicinal systems (e.g., Chinese herbal medicines) also face quality control challenges, including high heterogeneity in cultivation environments, lack of standardization in processing techniques, and significant batch‐to‐batch variations in active ingredients. The absence of these standardized systems not only limits the clinical application of natural products but also poses substantial difficulties for in‐depth analysis of their pharmacodynamic mechanisms.

Similar to all bioactive substances, natural products may exert pharmacological effects and nonspecific off‐target effects on normal tissues when acting on the body [363]. While they show potential in the treatment of encephalopathy, this characteristic also implies potential toxicological risks and side effects. However, existing studies still lack systematic evaluation of the adverse reactions potentially induced by natural products and the risks of long‐term medication. Particularly, under the synergistic effects of multiple components, the mechanisms underlying their potential impacts on normal tissues of the body have not yet been clearly elucidated. The lack of such a systematic safety evaluation system has become a key barrier restricting the clinical translation of natural products.

Beyond the safety of individual natural products themselves, their combined application with chemical drugs also poses challenges. Existing studies have shown that the combined use of natural products and synthetic drugs may trigger complex pharmacokinetic interactions. A typical example is that the combined use of milrinone and fluoxetine may cause metabolic abnormalities and toxic risks in individuals with CYP2D6 functional deficiency or variation [364]. Therefore, there is an urgent need to conduct in‐depth research on the metabolic response characteristics of the body in the context of combined medication and establish personalized medication strategies based on pharmacogenomics, so as to provide a theoretical basis and practical guidance for clinical practice.

More importantly, the interspecies differences in neurobiological basis between rodents and humans make it difficult for disease models established based on rodents to accurately simulate the dynamic progression process of human encephalopathies. Specifically, existing models have obvious limitations in reproducing the progressive disease course, multietiological interactions, and complex clinical manifestations of human encephalopathies. In particular, they cannot fully reflect the associations between disease‐specific neurofunctional impairments and behavioral phenotypes. Such systematic biases in biological validity and clinical relevance hinder the translation efficiency of preclinical research findings into effective neurotherapeutic strategies, highlighting the urgency of developing novel model systems that more closely recapitulate the characteristics of human diseases.

## 9 Conclusion and Future's Prospects

In conclusion, future research should focus on establishing a clinical translation system for small‐molecule drugs represented by natural products. First, it is necessary to systematically promote multicenter, large‐sample randomized double‐blind controlled trials and establish a standardized efficacy evaluation system to scientifically verify their clinical value in the treatment of encephalopathies. On this basis, efforts should be made to actively explore synergistic treatment models of natural products and chemical drugs, and combined with adaptive clinical trial designs, systematically evaluate the advantages and applicable scenarios of combination therapies under strict monitoring.

To improve the targeting efficiency of natural products in the CNS, there is an urgent need to develop novel intelligent delivery strategies. The focus can be placed on constructing delivery platforms based on multifunctional nanocarriers, integrating BBB penetration technologies such as active targeting ligands and cell‐penetrating peptides, so as to break through the key bottleneck of their low bioavailability. Meanwhile, a standardized quality control system covering the entire chain from cultivation and processing to formulation should be established, laying a foundation for the reproducible application of natural products.

At the mechanistic research level, efforts should further integrate cutting‐edge technologies such as single‐cell sequencing and spatial multiomics, systematically elucidate the dynamic pathological processes of encephalopathies including SAE, DE, HE, and HIE, accurately identify key molecular events in disease progression, and thereby establish optimal intervention windows for drug therapy. On this basis, it is urgent to strengthen the construction and application of humanized model systems. For instance, by combining technologies like human organoids, induced pluripotent stem cell differentiation models, and gene editing, the key pathological processes of encephalopathy occurrence and development can be simulated, providing a solid theoretical basis for the optimization of personalized treatment strategies and precise intervention.

To summarize, regarding the pathogenic mechanisms and targeted therapeutic strategies of encephalopathies, there is an urgent need to conduct systematic and in‐depth exploration at the preclinical and clinical research levels. This will not only provide critical theoretical support and translational pathways for drug development represented by small‐molecule natural products but also is expected to accelerate the progression of promising therapeutic strategies toward clinical practice.

## Author Contributions

Shimeng Lv: writing – original draft. Xia Zhong: writing – review and editing. Ruirui Shang: writing – review and editing. Linghui Kong: writing – review and editing. Yufei Huang: writing – review and editing. Yuexiang Ma: writing – review and editing. Jing Teng: writing – review and editing. Sheng Wei: writing – review and editing. All authors have read and approved the final manuscript.

## Funding Information

This study was supported by the National Natural Science Foundation of China (no. 82274383), 2025 Shandong University of Traditional Chinese Medicine Graduate Quality Improvement and Innovation Program (YJSTZCX2025020), High Level Key Disciplines of Traditional Chinese Medicine: Basic Theory of Traditional Chinese Medicine, National Administration of Traditional Chinese Medicine (no. zyyzdxk‐2023118), the Special Funding for Taishan Scholars Project (no. tsqn202211137), the Chinese Medicine and Brain Science Youth Scientific Research Innovation Team, Shandong University of Traditional Chinese Medicine (no. 22202101), and the Postdoctoral Fellowship Program of CPSF under Grant Number GZB20240036.

## Ethics Statement

The authors have nothing to report.

## Conflicts of Interest

All authors declare no conflicts of interest.

## Data Availability

The authors have nothing to report.
